# Self-organized criticality in cortical assemblies occurs in concurrent scale-free and small-world networks

**DOI:** 10.1038/srep10578

**Published:** 2015-06-01

**Authors:** Paolo Massobrio, Valentina Pasquale, Sergio Martinoia

**Affiliations:** 1 Neuroengineering and Bio-nano Technology Lab (NBT), Department of Informatics, Bioengineering, Robotics, System Engineering (DIBRIS), University of Genova, Genova - Italy; 2 Department of Neuroscience and Brain Technologies, Istituto Italiano di Tecnologia (IIT), Genova - Italy

## Abstract

The spontaneous activity of cortical networks is characterized by the emergence of different dynamic states. Although several attempts were accomplished to understand the origin of these dynamics, the underlying factors continue to be elusive. In this work, we specifically investigated the interplay between network topology and spontaneous dynamics within the framework of self-organized criticality (SOC). The obtained results support the hypothesis that the emergence of *critical states* occurs in specific complex network topologies. By combining multi-electrode recordings of spontaneous activity of *in vitro* cortical assemblies with theoretical models, we demonstrate that different ‘connectivity rules’ drive the network towards different dynamic states. In particular, scale-free architectures with different degree of small-worldness account better for the variability observed in experimental data, giving rise to different dynamic states. Moreover, in relationship with the balance between excitation and inhibition and percentage of inhibitory hubs, the simulated cortical networks fall in a critical regime.

The spontaneous activity originated by the interactions of neuronal assemblies is a peculiar feature of the vertebrate nervous system^1^. In the cortex, it is characterized by oscillatory patterns which span different frequencies or rhythms^2^, while in reduced neuronal systems, it is mainly characterized by a mixture of spikes and bursts lasting from tenths to hundreds of milliseconds^3^. Its analysis has revealed that cortical networks generate scale-free activation patterns called *neuronal avalanches*, supporting the evidence of criticality in the brain. Such experimental findings come from *in vitro* (acute and organotypic cortical slices^4^, and also dissociated cultures^5^) and *in vivo* experimental models (awake monkeys^6^, anesthetized rats^7^ and cats^8^), up to the human brain^9^.

Among the possible factors that could support critical dynamics, the interplay between functional critical states and topological features of cortical networks remains poorly understood. Experimental evidences *in vitro* show that mature cortical assemblies not necessarily fall into a critical regime, but can also show subcritical or supercritical states^5^,^10^. Both at *in vitro* and *in vivo* level, functional and structural networks show features typical of complex networks. It has been claimed that hippocampal circuits show a preferential scale-free (SF) functional connectivity demonstrating the existence of hubs which orchestrate the activity of the entire assembly^11^. Also the assembly activation usually observed in primary cortical cultures is likely to be sustained by a SF organization of network connectivity^12^. However, also functional small-world (SW) networks have been introduced to justify the complex dynamics of cortical assemblies. During their development, dissociated networks evolve toward a SW topology^13^, which is likely to support the concurrent emergence of functional segregation (associated to local clusters) and functional integration (provided by long-range connections), as it has been found in the brain^14^. Moreover, it has been pointed out that functional connections are predictive of structural ones^14^, and that the topological parameters of structural networks are also conserved in functional networks^15^.

In this work, we aim at investigating the effects of random and scale-free/small-world topologies on neuronal avalanches’ distributions through a computational model which reproduces the electrophysiological activity of large-scale cortical networks. As experimental system, we used *in vitro* neuronal cultures grown onto Micro Electrode Arrays (MEAs), recognized as a suitable biological model of neuronal network dynamics^16^,^17^. Here, we start from experimental evidences that cortical assemblies can display critical, sub-critical and super-critical dynamic states. Then, by means of theoretical modeling and extensive simulations, we prove that different topologies of connectivity determine different dynamic states by driving the network from sub-critical, to critical, up to super-critical states. In particular, by considering physiological boundaries, the obtained results underline the existence of a tight interplay between the observed dynamics and the underlying topology. Actually, random networks, which can potentially support the emergence of different dynamic states, only show super-critical dynamics in a physiological domain of their firing regime. On the other hand, scale-free and small-world architectures account for the variability observed in experimental data and the transition from sub-criticality to criticality is ruled by the degree of “small-worldness”. Interestingly, we also found that only a specific (i.e., physiological) balance between excitation and inhibition is capable to drive the network towards a critical dynamic state.

## Experimental Results

Multi-site electrophysiological measurements of cortical cultures were performed in the mature stage of the network development (after the 3^rd^ week *in vitro*) and analyses were carried out to characterize the different dynamic regimes (i.e., states). In the same way, the corresponding activity of simulated networks was analyzed.

### *In vitro* cortical ensembles display different dynamic states

**Figure 1:**
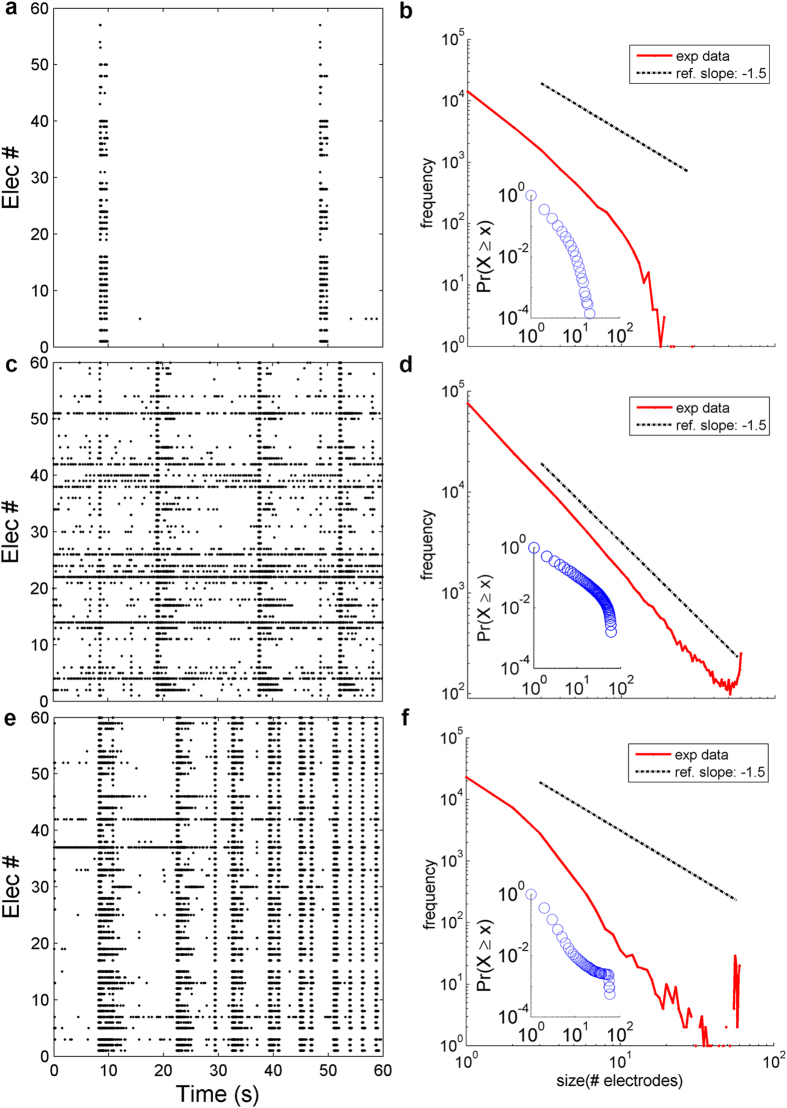
Experimental findings. **a**, **c**, **e**) Raster plots showing 1 minute of electrophysiological activity of three representative cortical cultures (27 DIV). **b**, **d**, **f**) Probability density functions (PDF) and the corresponding cumulative distribution functions (CDF) (inset) of avalanche sizes relative to the activity of the three networks of the left column.

In large-scale networks developing *ex vivo* and chronically coupled to MEAs (see Supplementary Information) neurons can freely form synaptic connections during development and, besides the fact that they must grow on a rigid substrate, they are not constrained by any additional external cues. These networks spontaneously exhibit complex spatio-temporal patterns of activity (see Supplementary Information), characterized by synchronized and distributed bursting activity mixed with highly variable spiking activity^18^. The raster plots of Fig. 1a,c,e show 60 s of spontaneous activity of three representative cortical cultures (27 *days in vitro*, DIV).

In Fig. 1b,d,f, we plotted the probability density function (PDF) and the cumulative distribution function (CDF) (inset) of avalanche sizes for the three cortical cultures (1-hour recordings) whose 1 min of activity is presented in Fig. 1a,c,e. In agreement with the seminal work of Beggs and Plenz^4^, neuronal avalanches were defined as sequences of consecutive active time bins, preceded and followed by a silent period. The optimal bin width was chosen for each recording/simulation according to the mean inter-event interval (IEI), as suggested in^4^ (see also Supplementary Information). Time bins were considered active when containing at least one spike on a single electrode/neuron. Avalanche size was then defined as the number of involved electrodes/neurons.

The obtained avalanche size distributions indicate a subcritical (Fig. 1b), a critical (Fig. 1d), and a supercritical regime (Fig. 1f). Criticality can be firstly assessed by fitting a power law *P*(*n*) ~ *n*^−*α*^ to the statistical distribution of avalanche sizes, where *n* is the avalanche size, *P*(*n*) is the probability of observing an avalanche of size *n* and α is the exponent of the power law (*α* > 0), giving the slope of the relationship (see also Supplementary Information). Sub-criticality can be associated to an exponential decay of the distribution of avalanche sizes, in the form *p*(*n*) ~ *e*^−λ*n*^, where λ is the corresponding exponent, indicating a dearth of bigger avalanches. Finally, super-criticality is inferred whenever there is an excess of avalanches involving the whole network with respect to the expected number according to a power-law distributed *P*(*n*).

Such three dynamic states correlate with remarkably different activity patterns (see the corresponding raster plots). By fitting the PDF of the representative critical culture by least-squares (LS) regression (see Supplementary Information), we estimated the power-law exponent to be equal to −1.7. By applying maximum likelihood (ML) estimation and rigorous statistical testing methods (see Methods) we found that such distribution is better described by a power-law truncated model, with a relative log-likelihood ratio test (LRT) (with a value of −1084.7, see Methods). This finding is compatible with the hypothesis of self-organized critical dynamics, since the exponential cut-off is observed in correspondence of the physical system size (in this case, the number of recording sites)^19^.

From the analysis of the spike trains, this variability in the exhibited regimes correlates with the level of synchronization of the network. Criticality was found in correspondence to an intermediate level of synchronization^5^. From this equilibrium condition, an increase or decrease of the network synchronization level drives the dynamics to a supercritical or subcritical regime, respectively^5^. By means of an *in silico* approach, we will explore the relationship between connectivity and dynamics to find whether peculiar topological configurations may better support such distinct dynamic states.

### Simulation Results

We simulated the dynamics of 1024 neurons (30% inhibitory) connected according to a SF and RND topology with 9 different configurations (labeled from Net_1 to Net_9) by increasing the average degree.

### Network topology characterization

**Figure 2:**
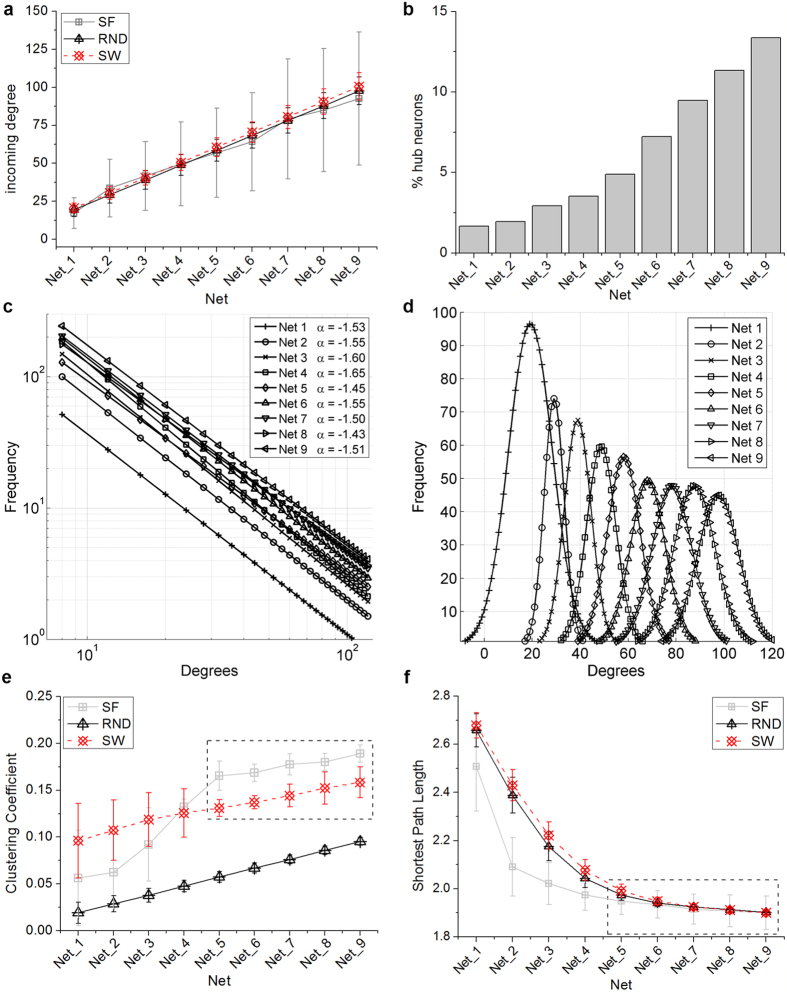
Network graph characterization. **a**) Incoming degree (mean ± standard error) for each topology as a function of the average number of connections (i.e. from Net_1 to Net_9). **b**) Percentage of hub neurons in SF networks. Degree distribution of **c**) SF and **d**) RND networks. **e**) Clustering coefficient and f) shortest path length for SF (grey), RND (black) and SW (red) networks. The dotted line box highlights SF networks which present small-world features.

For each network topology (i.e., RND, SF, SW), we generated 9 different configurations (labeled from Net_1 to Net_9), with an increasing average connectivity degree. Fig. 2a shows the incoming degree (mean ± standard error) for SF (grey), RND (black), and SW (red) networks. On average, all networks have a comparable incoming degree (similar considerations can be also done for the outcoming degree, data not shown); SF networks feature higher standard deviation values of the connectivity degree, given the presence of a small number of hubs. Although the mean of scale-free distributions with exponent *α *< 2 theoretically diverges, we decided to report the empirical mean value of degree obtained for scale-free networks together with other topologies for the sake of completeness. Fig. 2b quantifies the amount of hub neurons in SF networks, which span from 1.7% (for Net_1) to 13.4% (Net_9). The composition of hub neurons follows the same excitatory/inhibitory ratio of the entire network (i.e., 30% of inhibitory neurons). Figures 2c,d show the degree distributions of SF and RND networks, respectively. For all SF networks, the degree distribution can be fitted by a power-law (Fig. 2c) and the corresponding exponent lies between −1.65 and −1.43, with no specific correlation to the average degree. The degree distributions of RND networks have been fitted by a Gaussian distribution (Fig. 2d), whose mean value corresponds to the network average degree.

In the simulations presented in this work, we focused on the dynamics generated by only SF and RND networks. We generated SW network graphs only to make possible a comparison between the principal topological features (namely clustering coefficient and path length) of SF and RND networks with SW ones. In fact, their degree of “small-worldness”^20^ was evaluated by comparing for each generated SF and RND network the topological features with the corresponding SW one. As aforementioned, we compared the three different network topologies (for the same number of connections) by evaluating the clustering coefficient (Fig. 2e) and the shortest path length (Fig. 2f). As predicted by the theory, SF networks are always more clustered than RND ones. However, for low average degrees, the clustering coefficient of SW networks (red curves in Fig. 2e) is higher than that of SF ones. On the contrary, for higher average degrees than Net_4, SF networks present a higher (or comparable) degree of clustering than SW networks. We define such networks (i.e., from Net_5 to Net_9) as SF networks with small-world features (highlighted by the dashed box in Fig. 2e,f). We can also make similar considerations by looking at the path length (Fig. 2f): SF and SW networks reach comparable values of this parameter for high connectivity degree values. To preserve physiological features of *in vitro* cortical cultures, we introduced 70% of excitatory neurons and 30% of inhibitory ones^3^. The same percentages are approximately maintained by considering the total number of excitatory/inhibitory links both for SF and RND networks (see Supporting Information).

As presented in the Supplementary Information, functional connections are predictive of structural ones, and the topological parameters of structural networks are also maintained in functional ones (e.g., degree distribution). In light of this relationship, we could investigate the interplay between structural connectivity and critical dynamics in a computational model that mimics the firing and bursting statistics of *in vitro* cortical cultures as recorded by means of MEAs, and then extend such insights to experimental recordings where only functional connectivity maps can be derived.

SF and RND networks were simulated by modeling each node as an Izhikevich neuron (see Methods and Supporting Information). For each network, we simulated 10 realizations (10 minutes each) by changing the seed of the noise (modeled according to an Ornstein-Uhlenbeck process, see Methods) used to generate the spontaneous activity.

### Both random and scale-free networks show synchronized periods of activity

We first characterized the dynamics of SF and RND networks as a function of the degree by evaluating first-order statistics.

**Figure 3:**
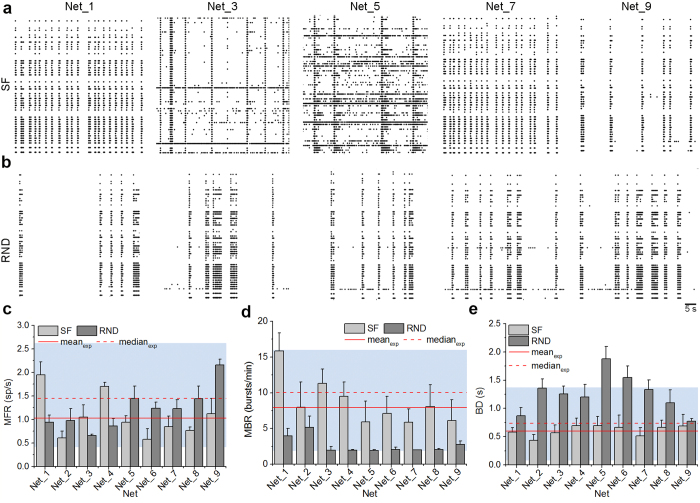
Simulated network dynamics. Raster plots of 60 s of simulated activity relative to five representative **a**) SF and **b**) RND networks, with an increasing level of connectivity (from Net_1 to Net_9). For the sake of clarity, only 60 neurons over 1024 (maintaining the excitatory/inhibitory ratio) are plotted. **c**) MFR, **d**) MBR, **e**) BD. Each bar represents the mean value over 10 realizations. Light and dark grey depict SF and RND networks, respectively. The light blue box represents the min-max values found experimentally, while the red dashed and solid line the median and the mean value (*n* = 10 cultures), respectively.

Figure 3 shows 60 s of activity of a subset (60 neurons) of representative SF (Fig. 3a) and RND (Fig. 3b) simulations by increasing the connectivity degree (i.e., from Net_1 to Net_9). All raster plots display a mix of spiking and bursting activity that summarizes what is typically found in experimental conditions. Quantitatively, Fig. 3c–e display the statistics of mean firing rate (MFR), mean bursting rate (MBR), burst duration (BD) over 10 realizations of each Net_*i*.

MFR statistics (Fig. 3c) over the different configurations show that, even by varying the average degree in a wide range (i.e. from Net_1 up to Net_9), the firing frequency lies in the range 0.5–2 sp/s (same range of experimental values). Additionally, neither an increase, nor a decrease can be identified by varying the overall connectivity degree. More interesting is what we obtained from the MBR and BD statistics (Fig. 3d,e). On average, all SF networks are more bursting than the corresponding RND ones, but with shorter bursts (mean value of about 0.6 s with respect to about 1.2 s). However, both SF and RND networks display a bursting dynamics compatible with the experimental findings (light blue box in Fig. 3c–e), both in terms of frequency and duration.

### SF networks promote sub- and critical dynamic states

We applied the avalanche detection algorithm (see Supplementary Material) to all simulations. Avalanches were detected by using as optimal time bin the average Inter-Event-Interval (IEI)^4^.

**Figure 4:**
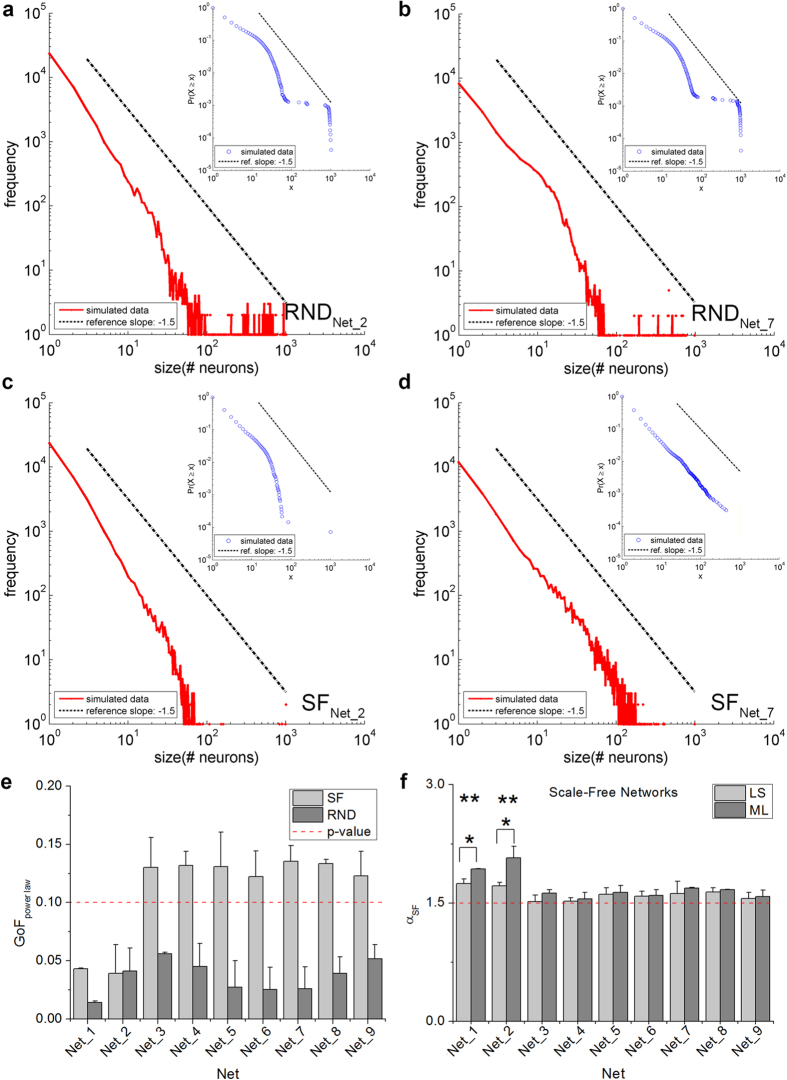
Avalanche size distributions and GoF evaluation. While RND networks lie in a super-critical state, SF networks pass from sub-critical (**a**) to critical states (**b**) by increasing the average connectivity degree (from Net_2 to Net_7). To help the reader visualize the results, all histograms include a dotted black line whose slope is −1.5. The insets show the avalanche size distributions evaluated in terms of CDF where *x* indicates the avalanche size. e, GoF evaluated for SF (light grey) and RND (dark grey) networks. The red dotted line indicates the *p*-level value used to assess power-law fitting. f, Comparison between power-law exponent values obtained with the LS (light grey) and ML (dark grey) fitting procedures for SF networks. Except for the configurations labeled as Net_1 and Net_2, the two methods produced equivalent results (not statistically different, ^*^p < 0.05) and close to −1.5 (red dotted line).

We evaluated the avalanche size distributions of all SF and RND networks. Figure 4 shows four significant examples of avalanche size distributions of two RND (a and b) and two SF (c and d) networks, respectively. Figure 4 illustrates that by varying in a wide range the connectivity degree of RND networks, the avalanche distribution always shows a peak in correspondence to the biggest avalanches after an exponential decay (Fig. 4a,b), indicative of a supercritical state^21^. On the contrary, in SF networks the dynamic state varies as a function of the average degree. Figure 4c shows the PDF of the avalanche sizes obtained from the simulation of a SF network with incoming average degree of 33.7 ± 19.0 (Net_2). The power-law behavior is not identifiable and an exponential drop can be observed after about 1 decade. By increasing the average degree up to 93.6 ± 43.7 (Net_7), the network shifts to a critical regime characterized by a linear relationship in the bi-logarithmic scale (Fig. 4d). Similar results can be also observed by looking at the CDF of avalanche sizes (insets of Fig. 4). To verify whether the power-law hypothesis is reasonable, given the data distribution, we used a *goodness-of-fit test* (see Methods). The Kolmogorov-Smirnov (KS) distance was used to measure the distance between the empirical CDF and the fitted model (by ML estimation). When the *p*-value is close to 1, the data set is considered to be drawn from the fitted distribution, otherwise it should be rejected. For our simulations, we set the significance threshold to 0.1^22^,^23^ (see Methods). *P*-values are higher than 0.1 for SF networks labeled from Net_3 to Net_9 (Fig. 4e). On the contrary, the avalanche distributions of other SF and of all RND networks present a *p*-value below 0.1 and thus cannot be considered as ruled by a power-law relationship (Fig. 4e).

Therefore, the first relevant result is that RND topologies, in the simulated physiological regimes, cannot support power-law avalanche size distribution, even by spanning the incoming average degree in a wide range (from 19.5 ± 4.4 to 97.7 ± 9.1). The second result is that SF networks exhibit a dual behavior: for lower connectivity degrees (Net_1 and Net_2), the fitting methods do not support a power-law model for the avalanche distribution; for higher connectivity and by increasing the average degree, the avalanche size distribution approaches a −1.5 power-law regime (Fig. 4e,f). We further confirmed the likelihood of the power-law fitting by comparing the results with the ones obtained by fitting other typical distributions to the data, namely truncated power-law, exponential and lognormal, by means of the LRT (see Supplementary Information). With this refinement, only avalanche size distributions from Net_6 to Net_9 are better fitted by a power-law model than by any other tested model (see Supplementary Information, Table S1). This suggests that criticality can be achieved only by SF networks with a relatively high average connectivity degree, and with a consistent degree of small-worldness.

### Proof of criticality

The proof that SF networks with SW features display power-law distributed avalanche sizes is not sufficient to assess that their dynamics is actually self-organized critical. In this work, we pursued two routes to check whether the behavior of these networks can be definitely considered self-organized critical, namely: network rescaling^4^ and characterization of the mean temporal profile of the detected avalanches^24^.

### Network rescaling

**Figure 5:**
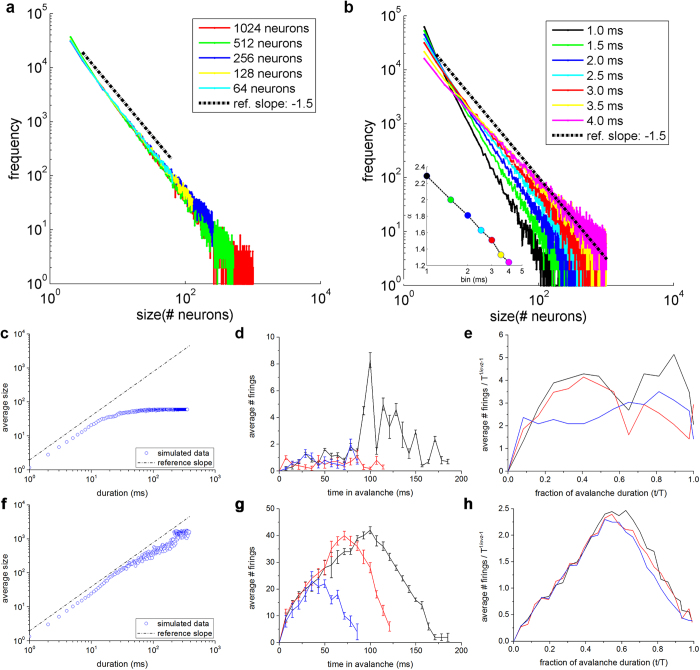
Proof of criticality. **a**) Avalanche size distribution (PDF) of a representative SF network (Net_7) by considering 1024 (red), 512 (green), 256 (blue), 128 (yellow), and 64 (cyan) neurons. **b**) Size distribution follows power-law distribution independently of bin width. The −1.5 exponent is obtained with a bin width equal to the average IEI (red line). Inset: Dependence of the slope *α* on the bin width. Average avalanche size vs duration of **c**) Net_2 and **f**) Net_7 (only one realization considered). The dot-dashed black line corresponds to power-law distributions with critical exponent equal to 1.3. Three representative avalanche shapes (blue, red, and black lines) for **d**) Net_2 and **g**) Net_7. Those shapes have been realized by averaging the temporal profiles of all avalanches with 3 different durations (85, 120, and 185 ms). Three representative examples of collapsed avalanche distributions relative to **e**) Net_2 and **h**) Net_7.

If those networks were actually self-organized critical, avalanche size distributions would follow the same power-law (same exponent) when varying the system’s size (i.e. number of neurons). In addition, the power-law should remain linear when avalanche sizes are measured using different time bin widths^4^. For this reason, we also tested if and how the number of considered neurons changes the slope of the avalanche distribution (Fig. 5a), and if the power-law is preserved by changing the bin width (Fig. 5b). To this purpose, we rescaled the network by halving in four steps the number of neurons from 1024 to 64 (Fig. 5a). Since in our model no spatial measure is taken into account, we only chose the discarded neurons by keeping constant the relative number of hubs.

The rescaling procedure shows that the power-law behavior of avalanche size distributions holds when varying the system’s size, and that the cut-off point is roughly correspondent to the total number of neurons, according to what experimentally found in^4^,^5^. In addition, by sweeping the bin width from 1.0 to 4.0 ms, we observed a linear relationship between the slope of the avalanche size distribution and the bin width itself (Fig. 5b inset). This is a further demonstration of the robustness of the power-law for avalanche sizes at multiple time scales. We also found that the −1.5 exponent is observed for a bin equal to 3.0 ms (red line), which approximately corresponds to the IEI of that network.

### Avalanche shape profile

One of the consequences of self-organized critical phenomena is that the mean temporal profile of events is universal across scales (data collapse). This means that for each avalanche of duration *T*, we can evaluate the average number of neurons (*s*) firing at time *t* as:
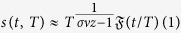
where 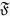 is a universal scaling function that rules the shape of the average temporal profile of avalanches. In the critical regime, the graphical representation of 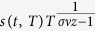 vs *t*/*T* for different values of avalanche durations *T* collapses onto the same universal scaling function 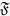. Mean field theory predicts that such a function belongs to the family of parabolas^25^. In Eq. (1), the critical exponent 1/*σνz* derives from the relationship between avalanche sizes and avalanche durations (Fig. 5c,f). Such exponent is related to the size (*α*) and lifetime (*τ*) critical exponents by the following equation^26^:
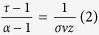


For the dataset of SF networks with different degrees of connectivity (i.e., from Net_1 to Net_9), we evaluated the relationship between avalanche size and duration (Fig. 5c,f), the avalanche shape profiles (Fig. 5d,g), and the collapsed shapes (Fig. 5e,h). For the sake of clarity, we plotted only data relative to one realization for each of the two representative SF networks, namely: Net_2 (top row of Fig. 5) and Net_7 (bottom row of Fig. 5). The same analysis was also performed for the other SF networks obtaining very similar results.

As reported in the previous section, the dynamics exhibited by Net_2 is subcritical (see Fig. 4c). The plots depicted in the first row of Fig. 5 confirm such results. Figure 5c shows the average avalanche size vs duration. The trend is far from that of a power-law distribution: the linear part lasts for less than one decade, and then saturates to a steady-state value. The consequences of such behavior can be also observed when looking at the avalanche shape. Figure 5d shows three representative temporal profiles for all avalanches with a specific duration (e.g., 85 (blue), 120 (red), and 185 (black) ms). The trend is quite irregular and no specific shape can be distinguished. Finally, the curves (obtained by rescaling the horizontal and vertical axes) of Fig. 5e do not collapse as expected and indicate a behavior far from criticality. On the contrary, in case of Net_7 (bottom row of Fig. 5) avalanche profiles and collapsed curves suggest a critical behavior. The average size vs duration curve (Fig. 5f) display a linear trend in log-log scale for more than two decades; the three average temporal profiles of avalanches of given duration (e.g., 85 (blue), 120 (red), and 185 (black) ms) recall a parabolic trend, which is confirmed in the collapsed shapes (Fig. 5h). A similar parabolic trend was also achieved for SF networks labeled as, Net_6, Net_8, Net_9, i.e., the ones that have been defined as critical.

### Synaptic weight distribution affects critical dynamics

**Figure 6:**
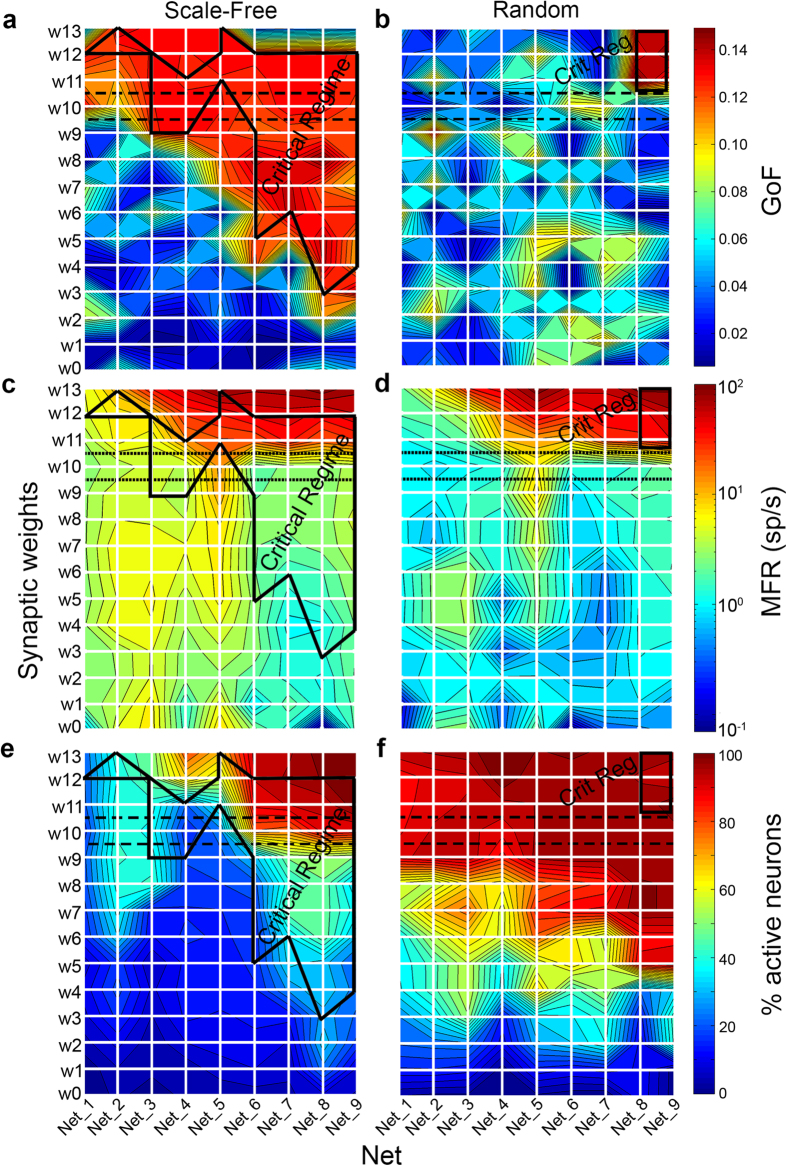
False color maps of GoF and activity parameters obtained for different synaptic weights’ average values and connectivity degree. GoF for **a**) SF, **b**) RND networks. MFR for **c**) SF, **d**) RND networks. Percentage of active neurons for **e**) SF and **f**) RND networks. The solid black polygon highlights the parameters’ domain that corresponds to a critical regime. The black dashed rectangle shows the default synaptic weight (i.e. used in the previous simulations).

In the previous simulations, we kept constant the synaptic weight distribution according to a Gaussian process, with a given mean and standard deviation (see Methods). To check whether synaptic weights affect criticality, we swept both the mean and the standard deviation of the Gaussian distribution relative to the excitatory connections. We first swept the mean of the synaptic weights in a wide range (from 0.01 to 100) for all SF and RND networks, keeping constant the standard deviation (*std*_*exc*_ = 1). Particularly, we were interested in checking whether RND networks could reach a critical state with weaker or stronger synaptic coupling. Figure 6 depicts three parameters, namely GoF (Fig. 6a,b), MFR (Fig. 6c,d), and percentage of active neurons (i.e., neurons with a MFR ≥ 0.1 sp/s, Fig. 6e,f) in a false color map as a function of the mean synaptic weight (*y*-axis) and the average degree (*x*-axis). A black dashed box highlights the mean synaptic weight that was used in all previous simulations. We delimited with a solid black line, the critical regime area obtained by thresholding the GoF and performing the LRT. SF networks (Fig. 6a) reach criticality in a wider sub-region of the parameters’ space with respect to RND ones (Fig. 6b) and a clear relationship between the two parameters is observed: the higher the connectivity degree, the lower the mean synaptic weight needed to reach criticality. However, for the highest weights, the firing rate increases to saturation values (up to tens of spikes per second^18^, Fig. 6c) and the percentage of active neurons goes up to 100% (Fig. 6e). For the lowest weights, firing rates are very low (around 1 spike/s) and the percentage of active neurons spans from 30 to 60%. The picture is different for RND topologies, which can reach critical states (Fig. 6b), but only for the highest synaptic weights and the highest connectivity degree (solid black rectangle in the upper right corner). Moreover, this parameters’ configuration lead all neurons to be active (Fig. 6f) with non-physiological firing rates (up to 100 spikes/s). Figure 6d shows, for the same weights and degree, the corresponding MFR values clearly indicating that for RND networks, the region highlighted as critical is characterized by mean firing rates higher than 50 spikes/s, that are not consistent with experimental MEA recordings^18^ and the average activity of some cortical regions^27^. On the contrary, in SF networks, the MFR within the critical region (Fig. 6c) oscillates in a physiological range (from 0.2 to 20 spikes/s). Additionally, for SF networks it is worth noting that the percentage of active neurons involved in the network dynamics is generally lower than for RND networks with similar average weights and connectivity degree. Figure 6e shows that, in the critical region, for SF networks, less than 40% of neurons are active during avalanches. As the simulations show, we can state that the mean value of the synaptic weight distribution affects the dynamic regime of the network but, at the same time, even by varying this parameter in a wide range, it is not possible to reach criticality with RND topology while maintaining a plausible firing regime.

Similarly, we swept the standard deviation of the synaptic weights distribution (from 0.0 to 2.2) for all SF and RND networks, keeping constant the mean to the default value (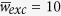), see Supplementary Information. In SF networks, the effect of increasing the standard deviation of the synaptic weight distribution is two-fold: first, it guarantees the presence of a critical dynamics, in correspondence of the high-connected networks; second, it provides a quasi-monotonic increase of the firing rate of the network. For the RND networks, the effect is more pronounced: the so obtained critical region by sweeping the standard deviation of the distribution of the synaptic weights of the RND networks is wider than the one obtained by sweeping the mean value (see Supplementary Information). However, also in this configuration, the obtained firing rate values present non-physiological values, although smaller than the ones of Fig. 6d (up to 80 spikes/s).

### Role of inhibition

We focused on the balance between excitation and inhibition with the aim at investigating its influence on the dynamics. Previously, we fixed a percentage of inhibitory neurons equal to 30%^3^. In this section, we checked whether by changing the ratio of inhibitory neurons and hubs we could enhance network synchronization or affect the critical regimes (i.e. change avalanche sizes’ distribution). By varying the proportion of inhibitory neurons, we also varied the global amount of inhibitory connections in the network (see Supporting Information), thus unbalancing the physiological ratio of excitation and inhibition found in cortical networks.

**Figure 7:**
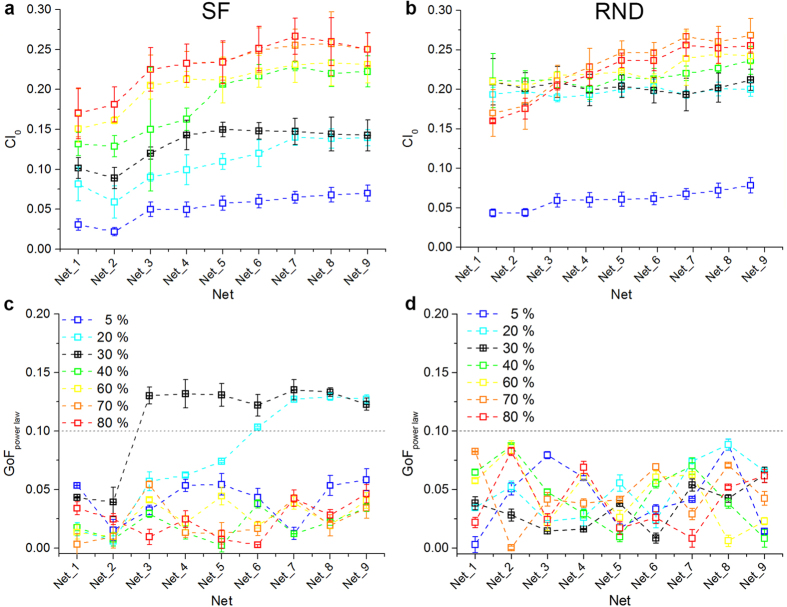
Intermediate excitation/inhibition ratios correlate with scale-free neuronal avalanches. **a**, **b**) *CI*_0_ of SF and RND networks with increasing percentage of inhibitory neurons (from 5 to 80%). **c**, **d**) GoF for SF and RND networks with increasing percentage of inhibitory neurons (from 5 to 80%). The grey dotted line indicates the *p*-level used to assess statistical significance of power-law fitting. While RND networks cannot achieve a critical state even when sweeping the percentage of inhibitory neurons in a wide range (from 5 to 80%), SF networks with an intermediate level of inhibition (20, 30%) lie in a critical state. Since all previously reported results were obtained from nets with 30% of inhibitory neurons, we used this value as reference (black trace).

Since power-law distributed neuronal avalanches in experimental data correlate with medium-level synchronization^5^, we measured such a feature by the coincidence index (*CI*_0_), namely the ratio of the integral of the cross-correlation function in a specified area (±1 ms) around zero to the integral of the total area (see Supporting Information). In Fig. 7a,b, we reported *CI*_0_ values for SF and RND networks respectively, featuring increasing percentage of inhibitory neurons (from 5 to 80%). By sweeping the percentage of inhibitory neurons we can observe that RND networks are much more correlated than SF ones for intermediate levels of inhibition (20, 30, and 40%) for all tested average degrees (i.e., from Net_1 to Net_9). This strong difference in *CI*_0_ decreases by lowering (5%) or increasing (70, 80%) the percentage of inhibitory neurons. With 5% of inhibition, the level of synchronization of the corresponding SF and RND networks is quite similar (Fig. 7a,b, blue traces), but always lower than that obtained for SF networks with 30% of inhibitory neurons. On the contrary, when inhibition is set to 70 or 80%, network synchronization strongly increases: both SF and RND networks (Fig. 7a, orange and red traces) are more synchronized than the SF reference networks, and also than RND ones starting from an intermediate degree (i.e., from Net_4).

SF networks with 70% of inhibitory neurons result the most synchronized, reaching on average higher values than RND networks. However, such increase of synchronization disrupts scale-free avalanche distributions. Figure 7c shows the power-law GoF for the different SF networks obtained by varying the percentage of inhibitory neurons. It can be noticed that power-law fitting is good only for intermediate percentages of inhibitory neurons (20 and 30%), i.e., values that guarantee medium-level synchronization (Fig. 7a). On the contrary, since RND networks are on average more synchronized than the corresponding SF ones, they are not able to sustain any critical regime (Fig. 7d).

We investigated in SF networks whether the hub composition (i.e., ratio between excitatory and inhibitory hubs, set at 30% in the previous simulations) could affect network synchronization, as well as the distribution of neuronal avalanche sizes. For all considered degrees (i.e. from Net_1 to Net_9), we simulated two possible configurations that we compared with the reference one (i.e. 30% of inhibitory hubs): i) SF networks with no inhibitory hubs; ii) SF networks with only inhibitory hubs.

**Figure 8:**
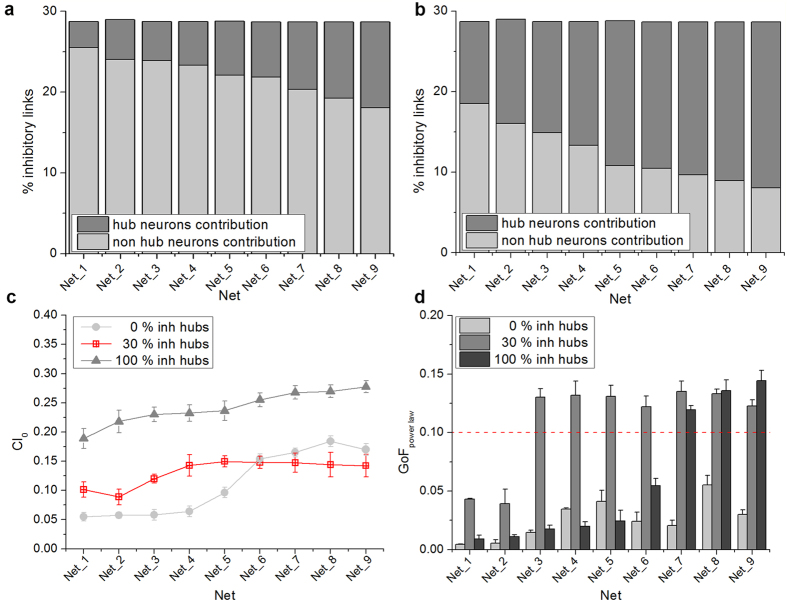
Effect of the hub composition in SF network dynamics. Percentage of inhibitory links taking into account the hub contribution for **a**) networks with 30% of inhibitory hubs and **b**) 100% of inhibitory hubs. **c**) *CI*_0_ of SF networks with an increasing percentage of inhibitory hubs. **d**) GoF for SF networks with an increasing percentage of inhibitory hubs. The red dotted line indicates the *p*-level used to assess statistical significance of power-law fitting.

Although we modified the hub composition, the global amount of inhibitory links remained unchanged (around 30%, Fig. 8a,b). In networks with 30% of inhibitory hubs, they contribute in a range that spans from 3% (Net_1) to 10% (Net_9) (Fig. 8a); otherwise, when all hubs are inhibitory, they contribute in a range from 10% (Net_1) to 20% (Net_9) of total inhibitory links (Fig. 8b). Figure 8c shows the synchronization level of networks with either only excitatory (circle, light grey) or only inhibitory (grey, triangle) hubs, compared to the standard condition where 30% of hubs are inhibitory (red, square). From these results we found that a strong inhibition in the hub composition makes the network more synchronized, with a trend similar to the one obtained for RND networks with 30% of inhibitory neurons (Fig. 8b). On the contrary, when only excitatory hubs are present, the synchronization level of the network is lower than the reference configuration (i.e. 30% of inhibitory hubs) for low connectivity degrees (i.e., until Net_5), whereas for Net_7, Net_8, and Net_9 we observed the opposite behavior. By varying the hub composition, we affected the dynamic states of the network. Figure 8d displays the GoF for SF networks in the case of no inhibitory (light grey bar), 30% inhibitory (grey bar), and 100% inhibitory (dark grey bar) hub neurons. The presence of only excitatory hubs does not allow the network to come to a critical state, since the GoF of the power-law fitting is always below the chosen significance level (Fig. 8d, light grey bars). On the contrary, with only inhibitory hubs, we observed two distinct behaviors. For networks with lower degrees of connectivity (i.e. from Net_1 to Net_6), the behavior is again not critical (Fig. 8d, dark grey bars, *p-value *< 0.1); when the average degree increases (i.e. from Net_7 to Net_9), criticality is achieved (Fig. 8d, dark grey bars). The computed exponents are equal to 1.52 ± 0.05, 1.48 ± 0.03, and 1.51 ± 0.04 for Net_7, Net_8, and Net_9, respectively. Finally, we point out that critical behavior, being intrinsic of a specific network topology, can be disrupted by changing the strengths of synaptic connections by emulating the delivery of GABA_A_-receptor antagonist (see Supplementary Material). Similar results have been observed experimentally while blocking inhibitory synapses by means of bicuculline and hence perturbing the excitation/inhibition network balance^28^.

## Conclusions

We studied the interplay between the emergence of different dynamic states (i.e. sub-critical, critical, super-critical)^5^ and the underlying network topology, taking as reference experimental model *in vitro* cortical assemblies. Few computational studies have been devoted to investigate possible relationships between neuronal avalanches and network architecture^29^, and most of these models are rather abstract and present poor biological accuracy/plausibility^30^,^31^. First, we found that both RND and SF networks display a mixture of synchronous bursts and asynchronous spikes activity usually observed in sensory-deprived *in vitro* preparations^32^,^33^. However only SF networks with SW features display either sub-critical (i.e. showing faster exponential decay) or critical (i.e. power-law) distributions of avalanche sizes depending on the level of small-worldness (correlated with network degree). Intuitively, in SW networks the coexistence of short- and long-range connections, promoting both segregation and integration of information through high clustering and short path length, should favor the generation of avalanches of all sizes, and thus a critical behavior^24^. On the other hand, in RND networks, where integration of activity prevails and clustering is low, only a supercritical behavior emerges at plausible (i.e., physiological) mean firing rates. Additionally, highly connected SF networks with SW features are less synchronized than the corresponding RND ones, thus allowing to achieve criticality. As witnessed by experimental findings^12^, a SF topology of connectivity plausibly underlies synchronous network patterns in living cortical networks, and hub cells are specifically involved in the generation of spontaneous network activations. These findings justify our choice suggesting that the combination of SF and SW properties is the necessary substrate for sustaining the network in an “optimal” regime of synchrony and permitting a smooth transition between dynamic states (from sub-criticality to criticality)^34^,^35^.

Finally, we showed that criticality is found at the edge of a phase transition^36^, tuned by varying the excitation/inhibition balance, and also the synaptic efficacy. In particular, as also shown in^37^, criticality is achieved when inhibition is approximately tuned at physiological values (20–30%), suggesting that pathologies causing impairment of inhibition mechanisms are likely to also disrupt criticality by inducing super-critical behaviors^38^. Recently, a few studies have been published trying to find a possible relation between the loss of criticality and brain diseases^39^, e.g. autism^40^. Those studies would likely provide new insights into the cellular and synaptic determinants of critical-like dynamics and structures in neural systems, and, at the same time, would help clarify the role of critical dynamics in normal brain functioning.

In perspective, it could be interesting to combine the study of the neuronal dynamics by means of the SOC theory with the chaos theory to see whether a possible correlation between criticality and presence/absence of chaotic behavior is achieved. In the literature, several studies demonstrate that complex neuronal networks can sustain different dynamic regimes depending on the underlying connectivity, (i.e., from totally ordered to chaotic behaviors^41^). Furthermore, it was also found that the same rule of connectivity can or cannot originate chaotic dynamics. Particularly, in the case of SW topologies, it depends on the probability of having short- and long-range connections^42^ or on the coupling strength and on the number of neurons of the assembly^43^. Finally, the high-performing computational capabilities of neuronal systems seem reachable neither in a chaotic, nor in an ordered dynamics, but in-between (*computation at the edge of chaos*)^44^. Following the SOC approach, in 2006 Chialvo came to a similar conclusion, saying that the computational properties of the brain are explained because it is “located at the border of an instability”^45^.

## Methods

### Neuron Model

The neuron model is based on the Izhikevich equations^46^. The possibility to obtain several spiking and bursting patterns depends on the choice of the four parameters of the neuron model^47^. In this work, we modeled excitatory and inhibitory populations of neurons with two different families of neurons: the family of regular spiking neurons (RS) and the family of fast spiking neurons (FS), respectively. Regular spiking neurons fire with a few spikes and short inter-spike-interval (ISI) at the onset of a stimulation. Differently, fast spiking neurons exhibit periodic trains of action potentials at higher frequencies without adaptation. Each neuron receives two inputs: the synaptic current from the other neurons, and a noisy current to model the spontaneous subthreshold electrophysiological activity. It is a stochastic source of noise, modeled according to an Ornstein-Uhlenbeck process:
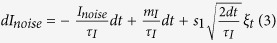


In Eq. (3) the quantity *ξ*_*t*_ is a white noise with zero mean and unitary variance. In this way, *I*_*noise*_ is Gauss-distributed at any time *t* and, after a transient of magnitude *τ*_*I*_ (correlation length), converges to a process with a mean equals to *m*_*I*_ and standard deviation *s*_*I*_. We set *τ*_*I*_ = 1 ms, *m*_*I*_ = 25 pA, and *s*_*I*_ = 9 pA.

The percentage of excitatory and inhibitory neurons was set, if not differently specified, to 70% and 30% respectively to be in the physiological range found in *in vitro* cortical cultures^3^.

### Network Models

The structure of the connectivity of a network is described by its adjacency matrix. All auto-connections are avoided. Then, the value of the non-zero *a*_*ij*_ elements of the adjacency matrix was multiplied by its corresponding synaptic weight. Excitatory/inhibitory synaptic weights were chosen from two normal distributions (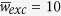, *std*_*exc*_ = 1; 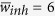, *std*_*inh*_ = 1). During simulation, synaptic weights were kept constant. To characterize the so generated graphs, we evaluated the path length (*L*), the clustering coefficient (*C*), and the connectivity degree (*D*) (see Supplementary Information).

Specific graphs with specific topologies were then created to model the connectivity, namely: random (RND)^48^, scale-free (SF)^49^, and small-world (SW)^50^.

#### Random Network

The fundamental assumption of random networks is that, despite the random placement of links, the correspondent graph is characterized by a uniform connection probability and a Gaussian degree distribution:
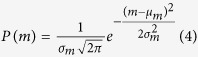


In Eq. (4), *m* is the degree, *μ*_*m*_ and *σ*_*m*_ are its mean and standard deviation values, respectively. With this definition, all nodes have roughly the same degree and we can define such architecture as single-scale.

#### Scale-Free Network

In SF networks^49^, the degree distribution follows a power-law: thus, if *m* is the number of edges which converge to a node (i.e. the connectivity degree), the power-law distribution is given by^51^:
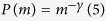


This law suggests that most nodes have just a few connections and others, named *hubs*, have a very high number of links. We define hubs those nodes whose degree exceeds a predefined threshold evaluated as:
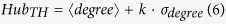
where <*degree*> and *σ*_*degree*_ represent the mean and the standard deviation of the degree distribution of the graph respectively and *k* is a multiplicative factor set at 4 in our simulations. Variations in the number of detected hubs are little (less than 10%) by sweeping *k* in a reasonable range (3 ≤ *k* ≤ 6).

#### Small-World Network

The small-world (SW) network model begins with a one-dimensional network made up of *N* neurons with connections between the *k* nearest and the next-nearest neighbors. Then, each link is rewired with a given probability *p* (i.e., shifting one end of the bond to a new node chosen at random from the whole system) with the constraint that no vertex can have a link with itself^50^. In the presented simulations, we set such a value to 0.5. It is worth noticing that similar results can be achieved when 0.4 ≤ *p *< 1.0. Within this range of rewiring probability, SF networks maintain small-word features.

### Fitting Procedures

To assess whether the distributions of avalanche sizes follow a power-law, we adopted/compared two different fitting procedures, namely: least-squares regression (LS) and maximum likelihood estimation (ML). Although the former has been widely used to estimate the power-law exponent by performing a linear regression on the log-log scale^4^,^5^, Clauset and co-workers^23^ showed that the logarithmic representation could lead to spurious power-law scaling induced by the stochastic nature of the phenomenon and LS fitting could potentially be biased and inaccurate in the case of power-law distributions. By following their approach we (i) fitted power-laws to empirical data by using ML estimation of the power-law exponent; (ii) we evaluated the GoF based on Kolmogorov-Smirnov statistics and likelihood ratios. These methods have been already used in the framework of neural data by other authors^22^,^52^.

We applied and compared both fitting procedures (i.e., LS and ML) to our simulations and we showed that these two methods gave equivalent results since the estimated exponents were not statistically different. Moreover, we evaluated the GoF by following the same procedures proposed in^23^ and we finally compared the power-law model accuracy with alternative distribution models (namely truncated power-law with exponential cut-off, exponential, and log-normal), by computing the LRT. Supplementary Information explains in detail the used alternative model distributions that were compared to the power-law.

## Additional Information

**How to cite this article**: Massobrio, P. *et al.* Self-organized criticality in cortical assemblies occurs in concurrent scale-free and small-world networks. *Sci. Rep.* **5**, 10578; doi: 10.1038/srep10578 (2015).

## Supplementary Material

Supplementary Information
