# Supplementary material for: Self-organized criticality in cortical assemblies occurs in concurrent scale-free and small-world networks
Source: Sci Rep. 2015 Jun 1;5:10578. doi: 10.1038/srep10578 (PMC4450754; doi:10.1038/srep10578)
Supplement: Supplementary Information [file srep10578-s1.pdf]

SUPPLEMENTARY INFORMATION

Self-organized criticality in cortical assemblies occurs in concurrent scale-free and small-world networks

Paolo Massobrio[1], Valentina Pasquale[2], Sergio Martinoia[1]

[1] Neuroengineering and Bio-nano Technology Group (NBT), Department of Informatics, Bioengineering, Robotics, System Engineering (DIBRIS), University of Genova, Genova - Italy.

[2] Department of Neuroscience and Brain Technologies, Istituto Italiano di Tecnologia (IIT), via Morego 30, 16163 Genova - Italy.

Materials and Methods

Computational Model

As mentioned in the main text, the neuron model is based on the Izhikevich equations[1]. This model depends on four parameters, which allow to reproduce the spiking and bursting behavior of specific types of cortical neurons. From a mathematical point of view, the model is described by a two-dimensional system of ordinary differential equations[1]:

$$\frac{dv}{dt} = 0.04v^2 + 5v + 140 - u + I_{tot} \tag{1}$$

$$\frac{du}{dt} = a(bv - u) \tag{2}$$

with the after-spike resetting conditions:

$$if\ v \geq 40mV \rightarrow \begin{cases} v \leftarrow c \\ u \leftarrow u + d \end{cases} \tag{3}$$

In Equations (1-3), v is the membrane potential of the neuron, u is a membrane recovery variable which takes into account the activation of K^+ and inactivation of Na^+ channels.

Equation (4) displays the used values for the four parameters.

$$a = \begin{bmatrix} 0.02 \\ 0.02 + 0.08r_i \end{bmatrix} \quad b = \begin{bmatrix} 0.2 \\ 0.25 - 0.05r_i \end{bmatrix} \quad c = \begin{bmatrix} -65 + 15r_i^2 \\ -65 \end{bmatrix} \quad d = \begin{bmatrix} 8 - 6r_i^2 \\ 2 \end{bmatrix} \tag{4}$$

In Eqs. (4), the first row relates to the excitatory (regular spiking, RS), while the second one to the inhibitory (fast spiking, FS) neurons. Although we used the class of RS and FS models, we introduced *per* each neuron a random variable r_i (which spans from 0 to 1) in order to introduce a further variability in the neuron dynamics: a RS neuron is obtained if $r_i = 0$, whereas if $r_i = 1$, a bursting neuron is obtained. Such distribution is biased towards RS neuron.

To model the morphological network connectivity, we made extensive use of graph theory[2]. Briefly, all graphs are composed by *nodes* which represent the neurons and *edges* which model the morphological connections among the neurons. If edges take into account the directionality of the connection (i.e., from a pre- to a post-synaptic neuron), the graph is named directed, otherwise it is called undirected. The structure of the graph is described by the adjacency matrix, a square symmetric matrix of size equal to the number of nodes N with binary entries. If the element $a_{ij} = 1$, a connection between the node j to i is present, otherwise $a_{ij} = 0$ means no connection. All the auto-connections ($a_{ii} = 1$) are avoided. Then, the value 1 of the non-zero a_{ij} elements are substituted with numbers representing the different synaptic weights drawn from a normal distribution. To model the dynamics of the network, each node of the graph is "replaced" by a neuron model, whose dynamics is simulated according to the Izhikevich equations (see Eqs. 1-2). Graphs are topologically

characterized by evaluating the path length (*L*), the clustering coefficient (*C*), and the connectivity degree (*D*).

A **path** is an ordered sequence of distinct edges and nodes that link a source node j to a target i: thus the path length L is the minimum number of distinct connections to reach i from j. The **clustering coefficient** $\boldsymbol{C_i}$ of a generic node i is evaluated as the ratio between the sum of the connections (with directionality) existing among the neighbors of node i and the sum of all nodes linked to i (without directionality). C_i lies between 0 and 1 and typically it is averaged over all nodes of the graph to obtain the mean clustering coefficient or the graph clustering coefficient.

The **connectivity degree,** or degree for short, $\boldsymbol{D_i}$ of a node i is the sum of all the incoming and outcoming connections. By averaging over the entire graph, we obtained the so called *average degree*.

A graph can be created according to different algorithms and can assume different properties in terms of the aforementioned metrics. Here below, we provided a description of the architectures used to model the connectivity of *in vitro* cortical networks, namely random (RND)[3], scale-free (SF)[4], and small-world (SW)[5].

Random Network. The model of RND network implemented in this work follows the original one devised by Erdõs and Renyi[3]. The fundamental assumption of random networks is that, despite the random placement of links, the correspondent graph is characterized by a uniform connection probability and a Poissonian degree distribution. The independent variables for building up a random graph are the number of nodes *N* and the total number of edges, with the condition that the minimum number of edges must be $N \cdot log\frac{N}{2}$. In its original formulation, the random algorithm was characterized by an evolution process, where, starting from *N* isolated nodes, subsequent random edges were added[6]. In our implementation, a random graph is chosen at random from the set of all symmetric graphs with *N* nodes and *m* edges.

Scale-Free Network. In SF networks[4] the degree distribution follows a power-law: thus, if *m* is the number of edges which incident to a node (i.e. the connectivity degree), the power-law distribution is given by[7]:

$$P(m) = m^{-\gamma} \tag{5}$$

where in neuronal systems the characteristic exponent γ usually lies between 1.3 (slice recordings [7]) and 2 (fMRI recordings [5]). To build a SF network, we made use of the algorithm described in[8], that has been proved to be efficient in terms of computation when dealing with large-scale networks. Nodes are added one by one successively. For each added node, *m* edges are generated,

where m is the minimum node degree. The endpoints are selected from the nodes whose edges have already been created, with a bias towards high degree nodes.

Small-World Network. The term small-world (SW) was coined by Milgram[9] with reference to social networks in which a person reaches any other person with a relatively short number of links (i.e., the so called "six degrees of separation" problem[9]). More recently, Watts and Strogatz[5] formalized and studied the features of this network topology. A SW graph lies in between a condition of regular lattice and randomness. In fact, by increasing the probability p of rewiring, the order of a regular lattice is disrupted, and when $p = 1$ a random graph is generated. Increasing the probability of rewiring, both the characteristic path length L and the clustering coefficient C decrease. In our case SW networks are obtained with a one-dimensional network made up of N neurons with connections between the k nearest and the next-nearest neighbors. Then, each link is rewired with a given probability p (i.e., shifting one end of the bond to a new node chosen at random from the whole system) with the constraint that no vertex can have a link with itself[5]. In the presented simulations, we set such a value to 0.5. It is worth noticing that similar results can be achieved when $0.4 \leq p < 1.0$.

If we consider RND, SF, and SW networks with the same average degree, the following relationships among the aforementioned metrics hold[6]:

$$C_{SW} \approx C_{REG} > C_{RND} \tag{6a}$$

$$L_{SW} \approx L_{RND} > L_{SF} \tag{6b}$$

where the subscript "REG" indicates a regular network. In this topology, each node is only connected to the neighbors and has the same number of links which implies a high degree of clusterization.

Cell culture

Dissociated cortical neurons were extracted from rat embryos and plated onto 60 planar TiN/SiN micro-electrodes (30 µm diameter, 200 µm spaced) at the density of 5-8 × 10^4 cells/device, which means about 1200 - 1400 cells/mm^2 (Fig. S1a). The procedure was approved by the European Animal Care Legislation and by the guidelines of the University of Genova. Micro-Electrode Arrays (MEAs) are coated with adhesion promoting molecules (poly-D-lysine and laminin). Neurons are maintained in culture dishes, each containing 1 ml of nutrient medium (i.e. serumfree Neurobasal medium supplemented with B27 and Glutamax-I) and placed in a humidified incubator having an atmosphere of 5% CO_2 at 37 °C. Further details can be found in[10].

Experimental setup and Electrophysiology

Electrophysiological activity was recorded during the third-fourth week *in vitro* to allow the maturation of the network[11]. The experimental set-up is based on the MEA60 System (Multi Channel Systems, MCS, Reutlingen, Germany) consisting of a mounting support with integrated 60 channels pre- and filter amplifier (gain 1200×) and a personal computer equipped with a PCI data acquisition board for real time signal monitoring and recording. The electrophysiological activity was recorded without any chemical or electrical stimulation (i.e., spontaneous activity). The recorded signals ranged from random spike activity to more complicated and synchronized burst signals, as depicted in Fig. S1b and c. Raw signals were recorded and sampled at 10 kHz, and data were then processed off-line by using a custom software to extract the spike trains (see Data Analysis).

Dataset

The dataset consists of n = 10 cortical cultures recorded for periods of at least 1 hour each, from 21 days *in vitro* (DIVs) to 35 DIVs.

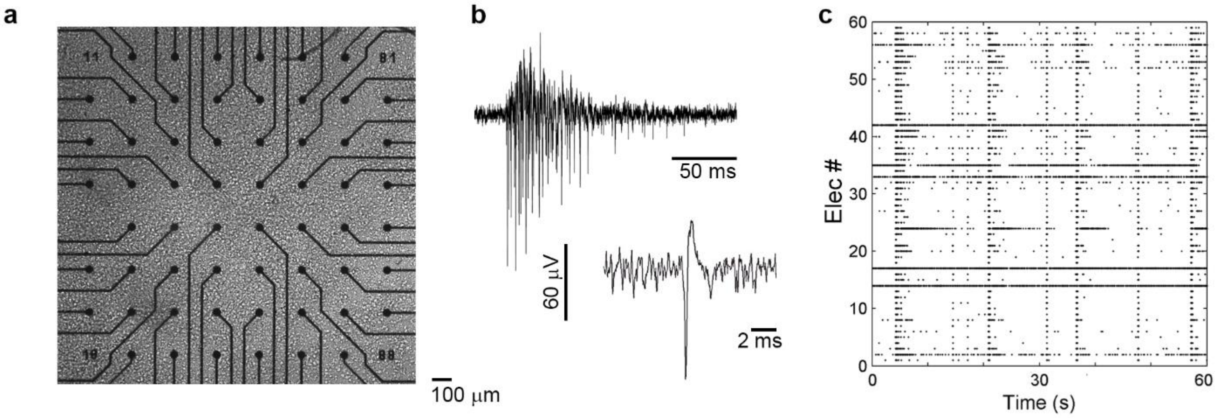

Figure S1. **MEA and electrophysiological signals overview**. **a**) Dissociated cortical culture at mature stage of development (27 DIV). **b**) Example of a burst (top) and a spike (down) recorded by one electrode. **c**) 60 seconds of spontaneous activity. Each row corresponds to a recording site, while each dot corresponds to a detected spike.

Data analysis

Experimental data were processed off-line by using a custom software package[12] developed in MATLAB (The Mathworks, Natick, MA, USA).

Spike detection. Extracellularly recorded spikes were detected using the PTSD (Precise Timing Spike Detection) algorithm[13]. Briefly, spike trains were built using three parameters: (1) a differential threshold set to 8 times the standard deviation of the baseline noise independently for each channel; (2) a peak lifetime period (set at 2 ms); (3) a refractory period (set at 1 ms). The data presented in the text were not spike sorted. This choice was made according to the fact that during a

burst a global increase of the activity produces a fast sequence of spikes with different and overlapping shapes which make the sorting difficult and unreliable[14].

Simulated data have been peak detected by means of a simple hard-threshold spike detection algorithm. In our simulations, we set the threshold value at 0 mV.

Burst detection. Both experimental and simulated data have been burst detected by means of the algorithms devised in[15]. Briefly, Detected bursts are sequences of spikes having an ISI smaller than a reference value (set at 100 ms in our experiments and simulations), and containing at least a minimum number of consecutive spikes (set at 5 spikes in our experiments and simulations).

For all the simulations, we first computed the mean firing rate (MFR), mean bursting rate (MBR), burst duration (BD), inter burst interval (IBI). Then, to characterize the interdependency between dynamics and connectivity, we focused on: (i) cross-correlation, to evaluate the degree of synchronization among the neurons; (ii) neuronal avalanches, to determine whether or not the system can be considered in critical state[16].

Cross-correlation, coincidence index. The cross-correlation (CC) function[17] was built by considering the spike trains (X and Y) of two neurons. It measures the frequency at which one cell fires (*target*) as a function of time, relative to the firing of a spike in another cell (*reference*) within a time frame around the spikes of the X train of $\pm T$ (T = 150 ms). Mathematically, CC is defined as:

$$C_{XY}(\tau) = \frac{1}{\sqrt{N_X N_Y}} \sum_{t_i=\left(\tau-\frac{\Delta\tau}{2}\right)}^{t_i=\left(\tau+\frac{\Delta\tau}{2}\right)} X(t_s)Y(t_s - t_i) \quad (7)$$

In Eq. (7), $\Delta\tau$ is the time shift or time lag (set at 1 ms) and t_s indicates the timing of an event in the X train. The corrected $C_{xy}(\tau)$ is obtained by means of a normalization procedure: each element of the array is divided by the square root of the product between the number of peaks in the X (N_X in Eq. 7) and the Y (N_Y in Eq. 7) train[18]. The CC function was evaluated by considering all pairs of neurons in the network. From the CC function, we extracted the coincidence index (CI_0) which is defined as the ratio of the integral of the CC function in a specified area (± 1ms) around the zero bin to the integral of the total area. This parameter allows to quantify the degree of synchronization of the neurons in the networks[10].

Inter-event interval (IEI). Considering the electrophysiological activity of the whole network, we derived the probability density of time intervals between successive spikes occurring at all the neurons, namely the inter-event interval (IEI) distribution[16]. Computing the average value of the IEI distribution, we obtained for every simulated network an estimate of the average time between two successive activations of any pair of neurons (Fig. S2). The average IEI was obtained by calculating the average value of the IEI distribution over the time interval [0, 100 ms]. The maximum value of the selected interval was determined as the average time interval corresponding to more the 99%

(99.87% for the SF and 99.52%, for the RND networks) of the area of the mean cross-correlogram (averaging cross-correlograms between all possible pairs of electrodes)[16].

Fig. S2a shows the IEI distribution (only the first 10 ms) for two representative realizations of Net_3. By averaging values over all network realizations, we derived the IEI mean values reported in Fig. S2b (mean ± standard deviation). It can be noticed how SF and RND networks show remarkable differences, especially at an intermediate range of connectivity degree (i.e., from Net_3 to Net_7), where the obtained values result statistically different ($p < 0.05$, Kruskal-Wallis non-parametric test). In this interval, SF networks present IEIs of about 3.5 ms, while the corresponding RND ones show IEI values of about 1.7 ms. On the contrary, at lower and higher degrees of connectivity, IEI values showed by the two topologies are not statistically different.

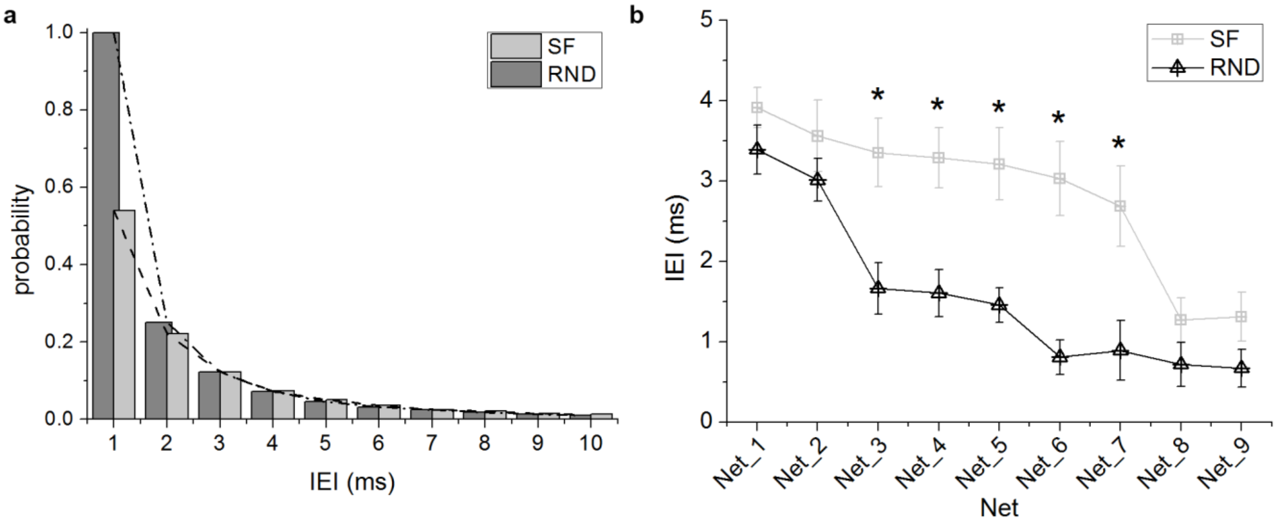

Figure S2. **Inter-event interval (IEI) distributions**. **a**) IEI distributions evaluated for two realizations of Net_3: SF and RND values are plotted in light and dark grey respectively. **b**) IEI of SF (grey) and RND (black) networks (mean ± standard deviation, *$p < 0.05$).

Avalanche detection. Following the pioneer works of Beggs and Plenz[16,19], a neuronal avalanche is defined as an event of widespread spontaneous electrical activity, preceded and followed by a silent period. Recordings are divided into time windows of duration Δt (called bins); inside each bin the spatial distribution of activity over the network represents a frame. A frame, which does not contain any spike, is called a blank frame. A neuron is active in a time bin Δt if, at least one spike is recorded, within that time window. Thus, a frame is active if, at least, one electrode is active. Following these definitions, a neuronal avalanche is a continuous sequence of active frames, preceded and followed by at least one blank frame (Fig. S3a). Without loss of generality, the time window used to bin the spiking activity was adjusted for each simulation according to the signal's timescale (i.e. average inter-event interval (IEI)).

Avalanches are usually characterized by their size: in this work, we defined the size of an avalanche as the number of neurons (or electrodes when experimental recordings are considered) being active

at least once within the avalanche. From the simulated spike trains, we computed neuronal avalanches and we estimated the probability density function (PDF) of avalanche sizes, as the relative frequency histogram, in which the height of each bar represents the proportion of avalanches of a given size. We also computed the complementary cumulative distribution function (CDF), which describes the probability that a random variable X with a given probability distribution is found at a value greater than or equal to x, (i.e., $P(x) = \Pr(X \geq x)$).

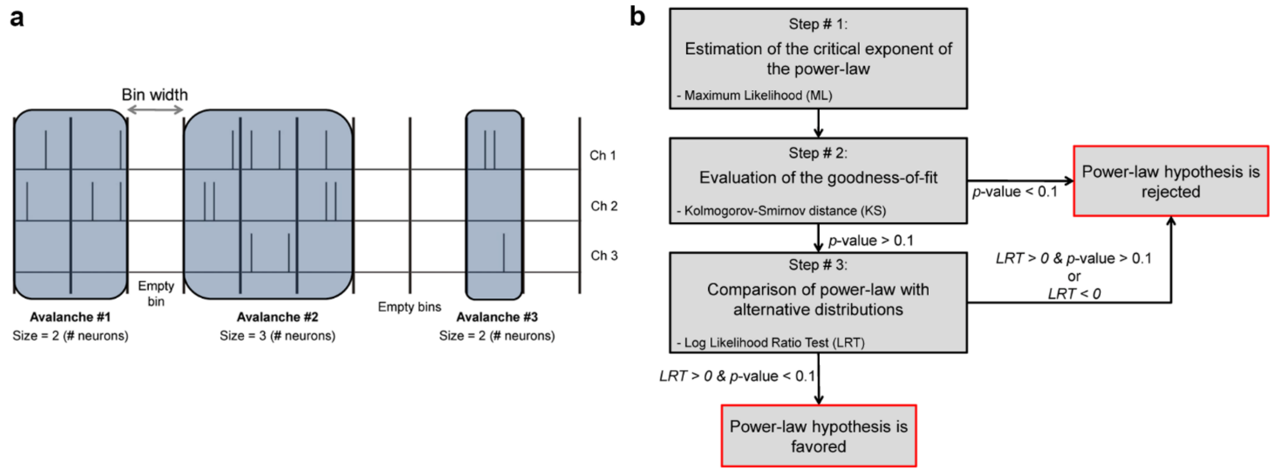

Figure S3**. Sketches illustrating the algorithms used to detect neuronal avalanches and perform power-law fitting assessment**. **a**) Avalanche detection algorithm. **b**) Fitting procedures used to assess the presence of a power-law distribution.

Model distributions

The statistically significance of the computed power-law distributions have been performed by following the procedures proposed by Clauset et al.[20]: after the evaluation of the goodness-of-fit, we compared the power-law model accuracy with alternative distribution models (namely power-law with exponential cut-off, exponential, and log-normal). Fig. S3b illustrates the aforementioned steps used to assess the presence of a power-law distribution. Here below, a description of the model distributions is provided.

Power-law. A continuous power-law distribution is described by a PDF $p(x)$ such that:

$$p(x)dx = Pr(x \leq X < x + dx) = Cx^{-\alpha}dx \quad (8)$$

where X is the observed value and C is a normalization constant.

In our case, x can only assume integer values (e.g. number of neurons), hence we must consider the corresponding discrete PDF of the form:

$$p(x) = \Pr(X = x) = Cx^{-\alpha} \quad (9)$$

In both cases, $p(x)$ diverges as $x \to 0$, so one can identify a lower bound x_{min} to the power-law regime. The normalizing constant C can be easily computed by solving $\int_{x_{min}}^{+\infty} p(x)\, dx = 1$

(continuous case) or $\sum_{x=x_{min}}^{+\infty} p(x) = 1$ (discrete case), providing $\alpha > 1$. In this study (and in general for empirical data), an upper bound x_{max} is also present and is given by the finite system size, i.e. the number of electrodes in the recording array[21]. Therefore, C will depend on all parameters α, x_{min} and x_{max}.

Finally, we also considered the CDF, defined to be $P(x) = \Pr(X \geq x) = 1 - \sum_{x=x_{min}}^{x} p(x) = 1 - \sum_{x=x_{min}}^{x} Cx^{-\alpha}$.

Exponential. The PDF for the exponential distribution, with parameter $\lambda > 0$ and normalizing constant C, is

$$p(x) = Ce^{-\lambda x} \quad (10)$$

Power-law with exponential cut-off. The power-law distribution with exponential cut-off is defined as:

$$p(x) = Cx^{-\alpha}e^{-\lambda x}$$

with $\lambda \geq 0$ and $\alpha > 1$. (11)

Log-normal. The PDF of the lognormal distribution is given by:

$$p(x) = C\frac{1}{\sqrt{2\pi}\sigma x}\exp\left[-\frac{(\ln x - \mu)^2}{2\sigma^2}\right] \quad (12)$$

with scale parameter σ and location parameter $\mu \geq 0$.

Parameters estimation

Least Squares Regression. The least squares (LS) regression considers directly a power-law model to the histogram of the avalanche distribution:

$$p(x) \propto x^{-\alpha} \quad (13)$$

In Eq. (13), x is the independent variable (e.g. avalanche sizes' occurrence frequencies) and α the exponent or scaling parameter. Taking the logarithm of both sides of Eq. (13), we can observe that the power-law distribution obeys $\ln p(x) = -\alpha \ln x + const$, implying that it follows a straight line on a bi-logarithmic plot. Thus, if a distribution approximately falls on a straight line, we can say that the distribution follows a power-law with an exponent equals to the slope of the straight line. Such slope is evaluated by performing least squares linear regression on the logarithm of the histogram.

In this work, we applied such method by excluding from the fitting the first bin, corresponding to avalanches of unit-dimension and the last bins of the histogram, corresponding to avalanches whose probability is less than 1‰ of the maximum value of the distribution.

Maximum Likelihood Estimation. Let x be the independent variable (e.g., avalanche sizes' occurrence frequencies) to which we wish to fit the power-law distribution reported in Eq. (13). A power-law distribution is described by the probability density function $p(x)$, reported in Eq. (8).

In Eq. (9), if $x \to 0$ the probability density $p(x)$ diverges: this means that not all the values of $x \geq 0$ can be taken into account. Thus, it becomes necessary to define a lower bound (x_{min}) for the power-law behavior. The ML method estimates first such x_{min} value, and then, the scaling parameter α of Eq. (13) by means of the two following relationships in the case of continuous and discrete case[20]:

$$\alpha = 1 + n\left[\sum_{i=1}^{n} ln\frac{x_i}{x_{min}}\right]^{-1} \quad (14a)$$

$$\alpha = 1 + n\left[\sum_{i=1}^{n} ln\frac{x_i}{x_{min}-\frac{1}{2}}\right]^{-1} \quad (14b)$$

where x_i are the observed values of x which satisfy the condition $x_i \geq x_{min}$.

To choose the best value of x_{min}, several strategies can be found in the literature[22]. The approach followed in this work is based on the work of Clauset and co-workers[23]. Practically, we consider the value of x_{min} that makes the probability distribution of the measured data and the best-fit power-law model as similar as possible above x_{min}. To measure the distance between the cumulative distribution function of the data and the fitted model, the Kolmogorov-Smirnov distance was used, since it can be used also for non-normal distributed data:

$$D_{KS} = \max_{x \geq x_{min}} |S(x) - P(x)| \quad (15)$$

In Eq. (15) $S(x)$ and $P(x)$ are respectively the cumulative distribution function of the observations under the condition $x > x_{min}$ and the power-law model which best fits the data in the interval $x > x_{min}$.

Goodness-of-Fit. After the estimation of the critical exponent and of the power-law fitting, we have to verify whether the power-law hypothesis is reasonable or not, given the data distribution. To this end, it becomes necessary the use of a *goodness-of-fit test* which generates a p-value that quantifies the likelihood of such hypothesis. This method works as follows: the fitted distribution is sampled to generate artificial data-sets; then the Kolmogorov-Smirnov distance between each data-set and the fitted distribution is evaluated, producing the distribution of Kolmogorov-Smirnov distances expected if the fitted distribution is the true distribution of the data. A p-value is calculated as the proportion of artificial data sets showing a poorer fit than fitting the observed data set. When this value is close to 1, the data set can be considered to be drawn from the fitted distribution, and if not, the hypothesis should be rejected. We chose a p-level equal to 0.1, meaning that the power-law hypothesis is ruled out if $p \leq 0.1$ according to[20,24].

Log-Likelihood Ratio Test (LRT). Although the Kolmogorov-Smirnov goodness-of-fit test provides results in favor of a power-law distribution, we would like to compare it with other likely distributions. To this end, we used the *log-likelihood ratio test* (LRT) statistics[20]. Practically, the likelihoods of two competing distributions are evaluated and compared as follows:

$$LRT = \ln \frac{L(power\ law)}{L(alternative\ distribution)} \tag{16}$$

where L indicates the likelihood of a distribution. If $LRT > 0$ the power-law model is preferred, otherwise it should be rejected. However, the sign of LRT is not sufficient to assess whether the power-law is the best model to the data, because of possible statistical fluctuations. Following the method proposed by Vuong and colleagues[25] to test the significance of the LRT, we computed the p-value: if it is small ($p < 0.1$) the power-law model can be considered as the best candidate to fit the experimental data. Fig. S3b summarizes the whole procedure.

Sub-critical distribution

The same procedure was applied to verify whether the cumulative distribution function of a sub-critical dynamics is ruled by an exponential distribution function. Following the previously described approach, we proceeded as follows.

Let x be the independent variable (e.g., avalanche sizes' occurrence frequencies) to which we wish to fit the exponential distribution. An exponential distribution is described by the probability density function $p(x)$ of the form:

$$p(x) = \Pr(X = x) = \begin{cases} \lambda_0 e^{-\lambda_0 x_j} & if\ x_j \in R_x \\ 0 & otherwise \end{cases} \tag{17}$$

where $R_x = [0, +\infty)$, and λ_0 is the parameter to estimate with ML by means of the following equation:

$$\lambda_0 = \frac{n}{\sum_{j=1}^{n} x_j} \tag{18}$$

Equation (18) shows that the estimation of λ_0 corresponds to the reciprocal of the mean.

The procedure to evaluate the goodness-of-fit of the exponential distribution follows the same steps used for the power-law one, namely: i) calculation of the Kolmogorov-Smirnov statistics and likelihood ratios (see Eq. 15); ii) c*omparison of the exponential accuracy with alternative distribution models* (power-law, exponential cut-off, and log-normal) with Eq. (16).

Additional Results

Excitatory/inhibitory links balance

The same percentages are approximately maintained by considering the total number of excitatory/inhibitory links for both SF and RND networks (Fig. S4a and b, black-square lines). By

sweeping the percentage of inhibitory neurons (5, 20, 40, 60, 70, 80%), also the percentage of inhibitory links follows the same proportionality rule (Fig. S4).

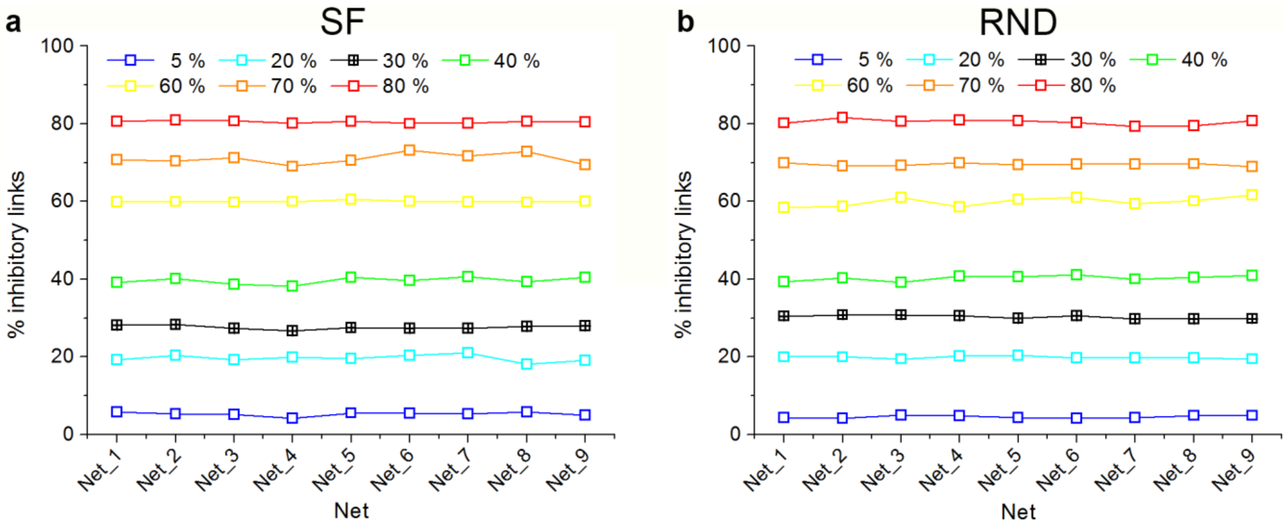

Figure S4. **Percentage of inhibitory links obtained by varying the percentage of inhibitory neurons** for **a**) SF and **b**) RND networks of increasing average degree (from Net_1 to Net_9).

Functional networks resemble structural features

We tested whether the structural features of the simulated networks are preserved in the functional networks inferred by means of the cross-correlation algorithm (see Supplementary Information, Data Analysis). For each realization of each SF and RND network, we applied the cross-correlation with time lag set to 1 ms and time frame set to 150 ms. After that, for each simulation we obtained an adjacency matrix containing the peak values of the cross-correlograms. Following the approach proposed in[26], we sorted all the statistically significant links based on the connection strengths, and we took into account only the ones that overcome an arbitrary threshold set as *mean* + 2 × *standard deviation* of the non-zero values of the cross-correlogram peaks.

For the obtained thresholded adjacency matrices, the degree distributions were evaluated and they are presented in Fig. S5a and b for SF and RND networks, respectively. Each colored line with symbols shows the average degree distribution computed over 10 realizations. It is rather evident that for SF networks, the degree distribution can be fitted with a power-law (Fig. S5a) with an exponent lying between -1.81 and -1.23. Interestingly enough, these exponents are not far from the corresponding ones obtained for the structural degree distributions (cf. Fig. S5c). On the other hand, as expected, the degree distributions of RND networks display a quasi-gaussian distribution, although some fluctuations can be observed.

Coherently with the results found by comparing diffusion imaging and resting state functional magnetic resonance imaging (fMRI) that show a tight relationship between structural and functional connections[27-29], also from our simulations we found that functional connections are predictive of

the structural ones, and that the topological parameters of structural networks are qualitatively preserved in functional networks.

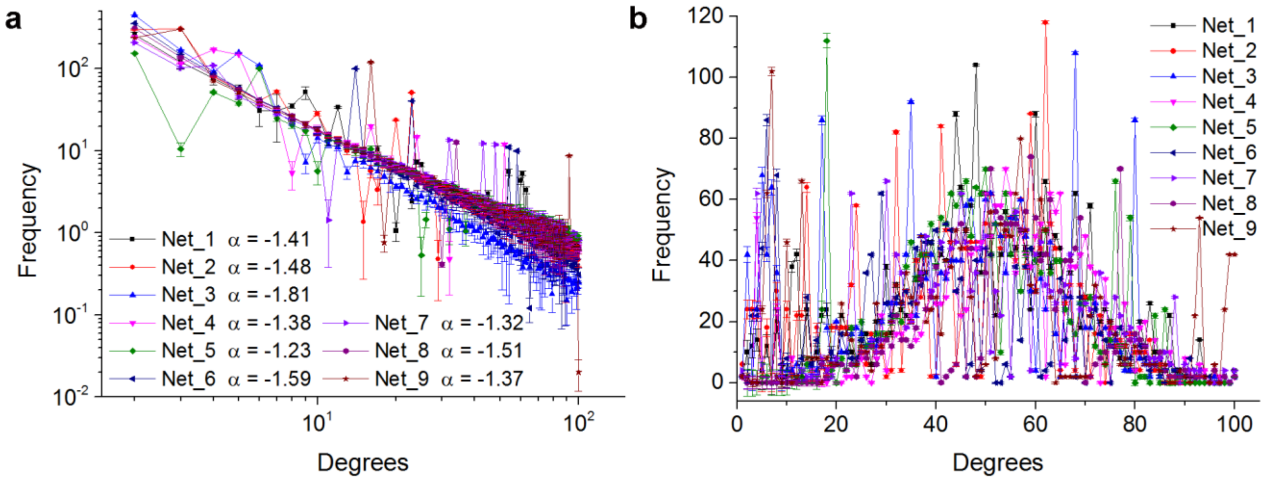

Figure S5. **Functional network characterization**. Degree distributions of **a**) SF and **b**) RND networks.

Distribution comparison

The plausibility of the power-law fitting can be estimated by comparing the fitting results with the ones obtained by fitting other similar data distributions, namely: truncated power-law, exponential and lognormal, by means of the *LRT*[20] (cf. Methods, Fitting procedures). Particularly, the exponential distribution is indicative of a sub-critical dynamic state; the log-normal distribution can be assimilated to a power-law because of its heavy-tail which makes it difficult to distinguish between the two; the truncated power-law distribution is indeed a power law distribution for finite-size systems (i.e., finite number of neurons). In the latter case, it is likely to observe an exponential cut-off above the system size.

Table S1. **Model comparison using LRT**. Power-law model is compared to truncated power-law, exponential, and lognormal alternative distributions. For each distribution, the LRT value and the *p*-value (bold case denotes statistical significance) are reported.

Net	GoF	Power Law vs Power Law truncated		Power Law vs Exponential		Power Law vs LogNormal		Support for Power Law
		LRT	*p*	LRT	*p*	LRT	*p*	
Net_01	0.043 ± 0.001	-	-	-	-	-	-	None
Net_02	0.039 ± 0.025	-	-	-	-	-	-	None
Net_03	0.130 ± 0.025	-1653 ± 453	-	5306 ± 304	$4.58 \cdot 10^{-3}$	2516 ± 192	$1.89 \cdot 10^{-2}$	None
Net_04	0.132 ± 0.012	-2423 ± 224	-	2344 ± 153	$9.25 \cdot 10^{-3}$	3672 ± 699	$1.30 \cdot 10^{-3}$	None
Net_05	0.131 ± 0.029	6423 ± 2324	$1.51 \cdot 10^{-1}$	4174 ± 2934	$1.30 \cdot 10^{-1}$	2962 ± 1923	$8.72 \cdot 10^{-3}$	Not significant
Net_06	0.122 ± 0.022	4423 ± 312	$\mathbf{1.53 \cdot 10^{-4}}$	3513 ± 168	$\mathbf{1.59 \cdot 10^{-3}}$	3507±137	$\mathbf{4.32 \cdot 10^{-4}}$	Good
Net_07	0.135 ± 0.014	6321 ± 762	$\mathbf{1.67 \cdot 10^{-4}}$	3212 ± 179	$\mathbf{3.92 \cdot 10^{-3}}$	3635 ± 112	$\mathbf{1.12 \cdot 10^{-4}}$	Good
Net_08	0.133 ± 0.004	6313 ± 226	$\mathbf{1.11 \cdot 10^{-4}}$	5818 ± 270	$\mathbf{3.84 \cdot 10^{-3}}$	2011 ± 539	$\mathbf{4.75 \cdot 10^{-4}}$	Good
Net_09	0.123 ± 0.021	4419 ± 983	$\mathbf{2.64 \cdot 10^{-4}}$	4346 ± 219	$\mathbf{2.20 \cdot 10^{-3}}$	2073 ± 199	$\mathbf{4.87 \cdot 10^{-2}}$	Good

For each simulated network, Table S1 shows the mean values of the LRT and the corresponding p-level for the alternative distributions.

The first two networks (i.e., Net_1, Net_2) were not considered since the goodness of the corresponding power-law fitting was not sufficient (cf. Fig. 4e of the main text). Net_3 and Net_4, although displaying a good power-law fitting, are better described by a truncated power-law model, as witnessed by the negative value of LRT, see Table S1. Net_5 displays positive LRTs when comparing power-law fitting with alternative models, but since the associated p-values exceed 0.1 the plausibility for a power-law distribution is not significant. Finally, avalanche size distributions from Net_6 to Net_9, are best fit by a power-law than by any other tested distribution, so they can be considered as ruled by a power-law. These results support the hypothesis that criticality is achieved by SF networks having a relatively high average connectivity degree, which also display, in turn, consistent small-world features.

Synaptic weight distribution affects critical dynamics

In Fig. 6 of the main text, we explored whether criticality is affected by the synaptic weigth distribution, in particular by sweeping the mean value of the excitatory connections. In this section, two further analyses have been performed: first, we swept the standard deviation of the synaptic weight distribution (from 0.0 to 2.2) for all SF and RND networks, keeping constant the mean to the default value ($\overline{w}_{exc} = 10$); second, only for the SF networks, we swept simultaneously both the standard deviation and the mean of the synaptic weight distribution for each degree (i.e., Net_i), and we computed the GoF.

The false color map of Fig. S6 (organized as the correspondent Fig. 6 of the main text), displays for SF and RND networks the GoF of the power-law distribution (Fig. S6a and b), the MFR (Fig S6a and b), and the percentage of active neurons (Fig. S6e and f) as a function of the standard deviation of synaptic weight (y-axis) and the average degree (x-axis). A black dashed box highlights the standard deviation of the synaptic weights that was used in all previous simulations. We delimited with a solid black line, the critical regime area obtained by thresholding the GoF and performing the LRT. By sweeping the standard deviation of the synaptic weights, two main considerations can be done. For SF networks, we observe that below the default value of the standard deviation (i.e., s5), we cannot achieve criticality independently of the degree (i.e., Net_i). The chosen value (s5) of the standard deviation guarantees also a good propagation of the electrophysiological activity of the network, as Fig. S6e shows: below s5, the percentage of active neurons is below 30%, while for values of standard deviation greater than s5, and with a medium/high level of degree (i.e., from Net_6), more than 80% of the neurons are active. Finally, as Figure S6c shows, we can see how the firing rate grows as a function of the standard deviation: the

greater is the standard deviation of the synaptic weight distribution, the higher is the value of the achieved firing rate.

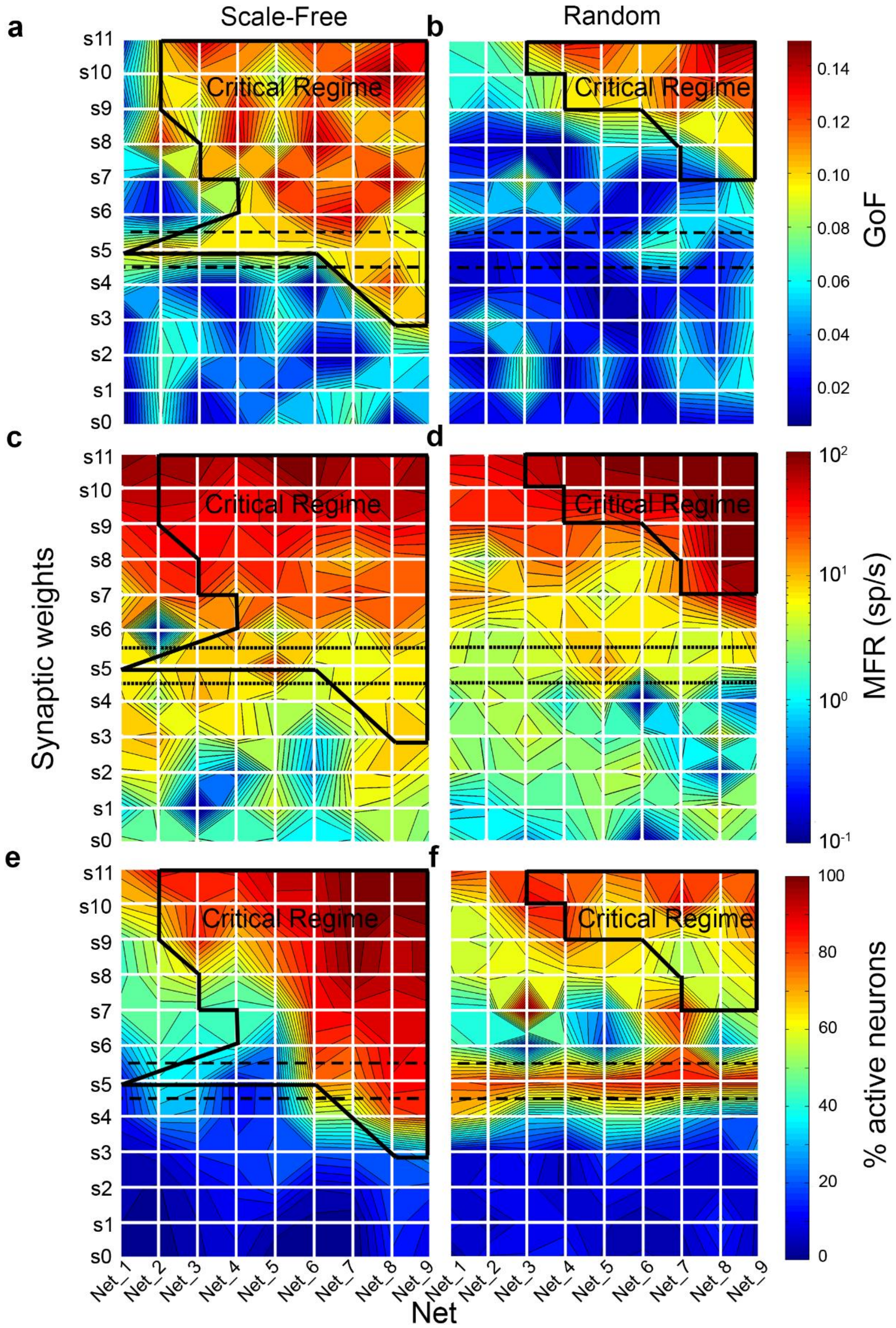

Figure S6. **False color maps of GoF and activity parameters obtained for different synaptic weights' standard deviation values and connectivity degree**. GoF for **a)** SF, **b)** RND networks. MFR for **c)** SF, **d)** RND networks. Percentage of active neurons for **e)** SF and **f)** RND networks. The solid black polygon highlights the parameters' domain that corresponds to a critical regime. The black dashed rectangle shows the default synaptic weight (i.e. used in the previous simulations).

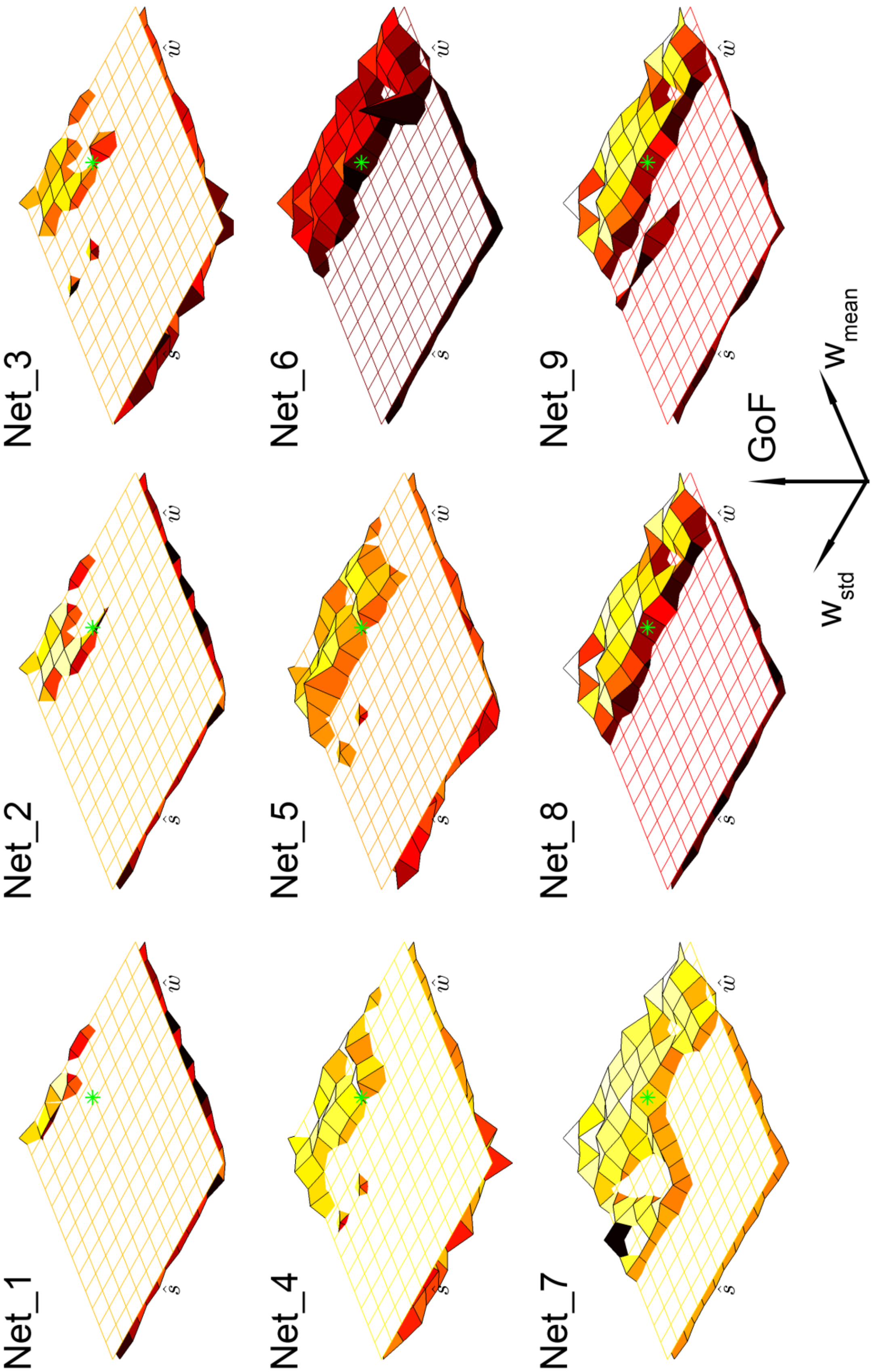

Figure S7. **False color maps of GoF for the SF networks.** Each 3D plot is relative to a network degree, and the different values of GoF are obtained by sweeping both the standard deviation and the mean of the synaptic weight distribution. The green asterisk (indicatingthe couple $\widehat{w}$, $\hat{s}$) highlights the default values.

For RND networks, the increase of the standard deviation of the synaptic weights induces a wider region of criticality than the one obtained by sweeping the mean value (Fig. 6b and S6b). However, also in this configuration, the obtained firing rate values present non-physiological values (Fig. S6d), although smaller than the ones of Fig. 6d (up to 80 spikes/s). The percentage of active neurons in RND networks displays a similar trend of the SF ones (Fig. S6f).

Finally, we simulated the emergent dynamics of each SF network (i.e., from Net_1 to Net_9) by sweeping simultaneously both the mean value and the standard deviation of the synaptic weight distribution, accordingly to the values used in Fig. 6 (w0-w13) and Fig. S6 (s0-s11). Figure S7 shows in a 3D fashion the GoF (*z*-axis) as a function of the mean (*y*-axis) and standard deviation (*x*-axis) of the synaptic weight distribution. The green asterisk highlights the default values used for the mean ($\hat{w}$) and the standard deviation ($\hat{s}$). The white-square planes identify the GoF threshold $p = 0.1$. Values greater than 0.1 suggest a critical behavior (cf. Methods, Fitting procedures).

From these simulations, two considerations emerge: (i) the degree of connectivity of the network is fundamental to push the network toward a critical state. As an example, considering the case of Net_1 of Fig. S7, an increase of both the mean and the standard deviation of the synaptic weight distribution does not guarantee a wide critical area, which can be easily obtained in Net_6, … Net_9. (ii) Comparing the effect of the mean and standard deviation of the synaptic weight distribution, it results that the mean is the most sensible parameter to move the network towards a critical regime.

Pharmacological manipulation

We tested if, by modifying the inhibitory synaptic transmission efficacy, without changing the composition of the network (i.e., 30% of inhibitory neurons) and the topology of the graph (i.e., SF networks with the same average degree), the power-law regime is preserved or not. By reducing the efficacy of all inhibitory connections (i.e. 50% and 100% of the original value) to mimic the effect of bicuculline (BIC), a supercritical behavior is achieved (Fig. S8b, grey and dark grey bars) for all the considered degrees.

The simulated effect of BIC was also evaluated in terms of network synchronization by means of the CI_0. Fig. S8a shows the trend of the CI_0 for all the three simulated conditions (i.e. spontaneous activity and different levels of BIC). These modeling results show the same qualitative trend of the experimental data[10], confirming that a supercritical state is typical of cultures with a high synchronization level, as induced by BIC stimulation.

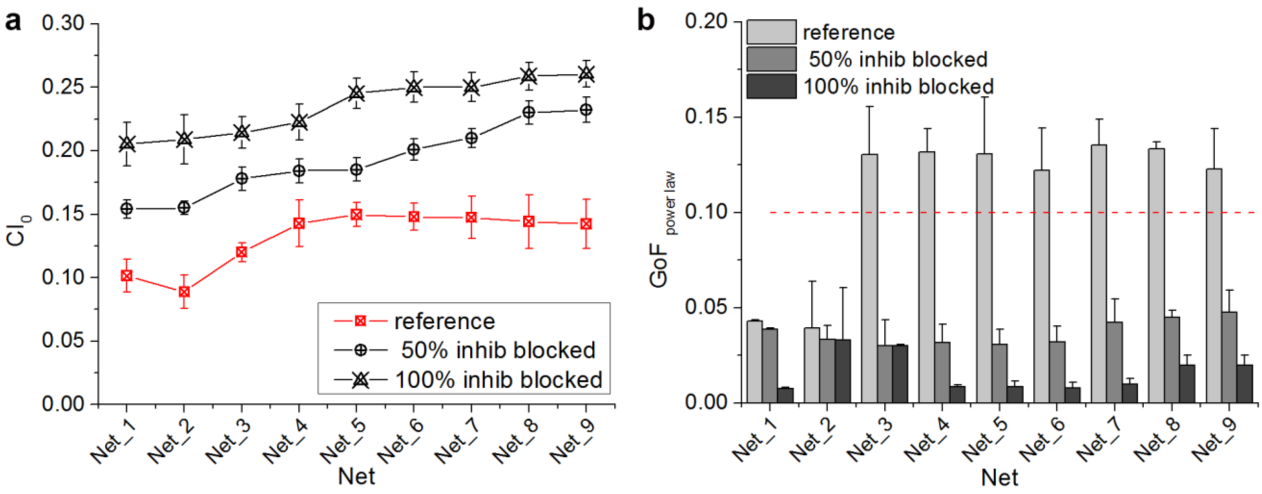

Figure S8. **Effect of BIC** on **a**) Coincidence Index, **b**) Goodness-of-Fit of the power-law model. Two levels of BIC concentration have been simulated in order to consider half-blocked (circle and grey bars) and fully-blocked (triangles and dark grey bars) inhibitory transmission.

Abbreviation List

Here below, we reported the abbreviations we used in both the main text and Supplementary Information.

bicuculline	BIC
burst duration	BD
clustering coefficient	C
coincidence index	CI_0
cross-correlation	CC
cumulative distribution function	CDF
days in vitro	DIV
degree	D
fast spiking	FS
goodness-of-fit	GoF
inter-event interval	IEI
inter-spike-interval	ISI
least-squares	LS
log-likelihood ratio test	LRT
maximum likelihood	ML
mean bursting rate	MBR
mean firing rate	MFR
Micro-Electrode Array	MEA
path length	L
probability density function	PDF
random	RND
regular spiking	RS
scale-free	SF
Self-Organized Criticality	SOC
small-word	SW

References

1 Izhikevich, E. M. Simple model of spiking neurons. *IEEE Trans. Neur. Net.* **6**, 1569-1572, (2003).

2 Sporns, O. in *Neuroscience Databases. A Practical Guide* (ed R. Kotter) (Klüwer, 2002).

3 Erdős, P. & Rényi, A. On random graphs I. *Publicationes Mathematicae* **6**, 290-297, (1959).

4 Barabasi, A.-L. & Albert, R. Emergence of scaling in random networks. *Science* **286**, 509-512, (1999).

5 Watts, D. J. & Strogatz, S. H. Collective dynamics of 'small-world' networks. *Nature* **393**, 440-442, (1998).

6 Albert, R. & Barabasi, A.-L. Statistical mechanics of complex networks. *Rev. Mod. Phys.* **74**, 47-97, (2002).

7 Dorogovtsev, S. & Mendes, J. Evolution of networks. *Advanced Physics* **51**, 1079-1187, (2002).

8 Batagelj, V. & Brandes, U. Efficient generation of large random networks. *Phys. Rev. E* **71**, 036113, (2005).

9 Milgram, S. The small world problem. *Psychol. Today* **2**, 60-67, (1967).

10 Pasquale, V., Massobrio, P., Bologna, L. L., Chiappalone, M. & Martinoia, S. Self-organization and neuronal avalanches in networks of dissociated cortical neurons. *Neuroscience* **153**, 1354-1369, (2008).

11 Wagenaar, D. A., Pine, J. & Potter, S. M. An extremely rich repertoire of bursting patterns during the development of cortical cultures. *BMC Neurosci.* **7**, (2006).

12 Bologna, L. L. *et al.* Investigating neuronal activity by SPYCODE multi-channel data analyzer. *Neural Netw.* **23**, 685-697, (2010).

13 Maccione, A. *et al.* A novel algorithm for precise identification of spikes in extracellularly recorded neuronal signals. *J. Neurosci. Methods* **177**, 241-249, (2009).

14 Wagenaar, D. A., Nadasdy, Z. & Potter, S. M. Persistent dynamic attractors in activity patterns of cultured neuronal networks. *Phys. Rev. E. Stat. Nonlin. Soft. Matter. Phys.* **73**, 051907, (2006).

15 Chiappalone, M. *et al.* Burst detection algorithms for the analysis of spatio-temporal patterns in cortical networks of neurons. *Neurocomputing* **65-66**, 653-662, (2005).

16 Beggs, J. M. & Plenz, D. Neuronal avalanches in neocortical circuits. *J. Neurosci.* **23**, 11167-11177, (2003).

17 Salinas, E. & Sejnowski, T. J. Correlated neuronal activity and the flow of neural information. *Nat. Rev. Neurosci.* **2**, 539-550, (2001).

18 Garofalo, M., Nieus, T., Massobrio, P. & Martinoia, S. Evaluation of the performance of information theory-based methods and cross-correlation to estimate the functional connectivity in cortical networks. *PLoS ONE* **4**, e6482, (2009).

19 Beggs, J. M. & Plenz, D. Neuronal avalanches are diverse and precise activity patterns that are stable for many hours in cortical slice cultures. *J. Neurosci.* **24**, 5216-5229, (2004).

20 Clauset, A., Shalizi, C. R. & Newman, M. E. J. Power-law distributions in empirical data. *SIAM Review* **51**, 661-703, (2009).

21 Klaus, A., Yu, S. & Plenz, D. Statistical Analyses Support Power Law Distributions Found in Neuronal Avalanches. *PLoS ONE* **6**, e19779, (2011).

22 Tierney, L., Kass, R. E. & Kadane, J. B. Fully exponential laplace approximations to expectations and variances of nonpositive functions. *Journal of the American Statistical Association* **84**, 710-716, (1989).

23 Clauset, A., Young, M. & Gleditsch, K. S. On the frequency of severe terrorist events. *Journal of Conflict Resolution* **51**, 58-87, (2007).

24 Touboul, J. & Destexhe, A. Can power-law scaling and neuronal avalanches arise from stochastic dynamics? *PLoS ONE* **5**, e8982, (2010).

25 Vuong, B. Q. H. Likelihood ratio tests for model selection and non-nested hypotheses. *Econometrica* **57**, 307-333, (1989).

26 Kanagasabapathi, T. T. *et al.* Functional connectivity and dynamics of cortical-thalamic networks co-cultured in a dual compartment device. *J. Neural. Eng.* **9**, (2012).

27 Honey, C. J., Kotter, R., Breakspear, M. & Sporns, O. Network structure of cerebral cortex shapes functional connectivity on multiple time scales. *Proc Natl Acad Sci U S A* **104**, 10240-10245, (2007).

28 Hagmann, P. *et al.* Mapping the structural core of human cerebral cortex. *PLoS biology* **6**, e159, (2008).

29 Bullmore, E. & Sporns, O. Complex brain networks: graph theoretical analysis of structural and functional systems. *Nat. Rev. Neurosci.* **10**, 186-198, (2009).
